# HssS activation by membrane heme defines a paradigm for two-component system signaling in *Staphylococcus aureus*

**DOI:** 10.1128/mbio.00230-24

**Published:** 2024-04-29

**Authors:** Vincent Saillant, Léo Morey, Damien Lipuma, Pierre Boëton, Marina Siponen, Pascal Arnoux, Delphine Lechardeur

**Affiliations:** 1 Université Paris-Saclay, INRAE, AgroParisTech, Micalis Institute, Jouy-en-Josas, France, Jouy-en-Josas, France; 2 Aix Marseille Univ., CEA, CNRS, BIAM, Saint Paul-Lez-Durance, France; Duke University School of Medicine, Durham, North Carolina, USA

**Keywords:** heme, *Staphylococcus aureus*, two-component system, virulence, membrane, biosensor

## Abstract

Strict management of intracellular heme pools, which are both toxic and beneficial, is crucial for bacterial survival during infection. The human pathogen *Staphylococcus aureus* uses a two-component heme sensing system (HssRS), which counteracts environmental heme toxicity by triggering expression of the efflux transporter HrtBA. The HssS heme sensor is a HisKA-type histidine kinase, characterized as a membrane-bound homodimer containing an extracellular sensor and a cytoplasmic conserved catalytic domain. To elucidate HssS heme-sensing mechanism, a structural simulation of the HssS dimer based on Alphafold2 was docked with heme. In this model, a heme-binding site is present in the HssS dimer between the membrane and extracellular domains. Heme is embedded in the membrane bilayer with its two protruding porphyrin propionates interacting with two conserved Arg94 and Arg163 that are located extracellularly. Single substitutions of these arginines and two highly conserved phenylalanines, Phe25 and Phe128, in the predicted hydrophobic pocket limited the ability of HssS to induce HrtBA synthesis. Combination of the four substitutions abolished HssS activation. Wild-type (WT) HssS copurified with heme from *Escherichia coli,* whereas heme binding was strongly attenuated in the variants. This study gives evidence that exogenous heme interacts with HssS at the membrane/extracellular interface to initiate HssS activation and induce HrtBA-mediated heme extrusion from the membrane. This “gatekeeper” mechanism could limit intracellular diffusion of exogenous heme in *S. aureus* and may serve as a paradigm for how efflux transporters control detoxification of exogenous hydrophobic stressors.

**IMPORTANCE:**

In the host blood, pathogenic bacteria are exposed to the red pigment heme that concentrates in their lipid membranes, generating cytotoxicity. To overcome heme toxicity, *Staphylococcus aureus* expresses a membrane sensor protein, HssS. Activation of HssS by heme triggers a phosphotransfer mechanism leading to the expression of a heme efflux system, HrtBA. This detoxification system prevents intracellular accumulation of heme. Our structural and functional data reveal a heme-binding hydrophobic cavity in HssS within the transmembrane domains (TM) helices at the interface with the extracellular domain. This structural pocket is important for the function of HssS as a heme sensor. Our findings provide a new basis for the elucidation of pathogen-sensing mechanisms as a prerequisite to the discovery of inhibitors.

## INTRODUCTION

*Staphylococcus aureus* is a Gram-positive opportunist bacterium that asymptomatically colonizes the skin and nostrils of nearly one-third of the human population (1). However, this organism can breach defensive barriers in the compromised host to cause invasive diseases such as endocarditis, toxic shock syndrome, osteomyelitis, and sepsis (1). Like most pathogens, successful infection by *S. aureus* involves the production of numerous virulence determinants including toxins, immune-modulatory factors, and exoenzymes and requires expression of factors that facilitate adaptation to the varied host environments (2–4).

Heme, an iron-containing tetrapyrrole, is the bioactive cofactor of blood hemoglobin (Hb) (5) (in this report, heme refers to iron protoporphyrin IX regardless of the iron redox state, whereas hemin refers to ferric iron protoporphyrin IX). The importance of heme resides in the unique properties of its iron center, including the capacity to undergo electron transfer, perform acid-base reactions, and interact with various coordinating ligands (5, 6). On the other hand, redox reactions of heme iron with oxygen generate reactive oxygen species (ROS), which provoke damage to proteins, DNA, and lipids (5, 6). Since heme is hydrophobic and cytotoxic, its concentration and availability must be tightly regulated.

In addition to utilizing heme as a nutrient iron source, *S. aureus* can employ both endogenously synthesized and exogenously acquired heme as a respiratory cofactor (7–9). Heme toxicity is offset in *S. aureus* by a conserved strategy for heme detoxification and homeostasis involving a heme-regulated efflux pump (HrtBA; Heme-regulated transport). HrtBA was also identified in *Enterococcus faecalis*, *Lactococcus lactis, Streptococcus agalactiae*, *Bacillus anthracis*, and *Corynebacterium diphtheriae* (10–13). HrtB topology classifies this permease as a MacB-like ABC transporter (14, 15). Rather than transporting substrates across the membrane, MacB couples cytoplasmic ATP hydrolysis with transmembrane conformational changes to extrude substrates from the periplasmic side or the lateral side of its transmembrane domains (TM) (15, 16). A heme-binding site was recently identified in the outer leaflet of the HrtB dimer membrane domain from which heme is excreted (17).

HrtBA expression in numerous Gram-positive pathogens is managed by HssRS (Hss; heme sensing system), a two-component system (TCS) (13, 18–20). HssS senses heme presence in the environment and transduces the signal to HssR, the transcriptional activator of *hrtBA* (13, 19–21). HssS is a prototypical family A histidine kinase (HisKA) with a short N-terminal cytoplasmic domain, and two TM helices flanking a 133 amino acid (AA) extracellular domain (19). The Ct cytoplasmic domain is organized in structurally conserved modules: the HAMP domain (present in histidine kinases, adenylate cyclases, methyl accepting proteins, and phosphatases) connects the second TM to the dimerization and histidine phosphorylation domain. A catalytic and ATP-binding (HATPase c) domain lies at the carboxyl terminus. Upon activation, HssS undergoes autophosphorylation of the His-249 residue and subsequently transfers the phosphoryl group to the Asp-52 residue of the HssR response activator (20, 21). The role of HssS phosphatase activity in the histidyl-aspartyl phosphorelay remains to be explored (22, 23). As HssS is activated by environmental heme, it is assumed that the histidine kinase (HK) extracellular domain (ECD) harbors the sensing function.

Here, we show that membrane, rather than extracellular heme, could trigger transient activation of HssS in *S. aureus*. To identify a domain within HssS that may participate in heme signal reception, we performed a structural simulation of the dimer that was docked with heme. A single-conserved hydrophobic structural domain (per monomer) with two conserved anchoring arginines at the interface between the membrane and the extracellular domain was predicted to accommodate heme. Based on this approach, we performed targeted mutagenesis and identified pivotal residues required for HssS sensing function and heme binding. Our work reveals a new mechanism of direct ligand sensing of a histidine kinase at the membrane level. We conclude that membrane heme control of HssS combined with membrane heme extrusion by HrtB constitutes a defense system for bacteria when they are exposed to lysed erythrocytes.

## RESULTS

### *hrtBA* induction is the readout for HssS activation

**Fig 1:**
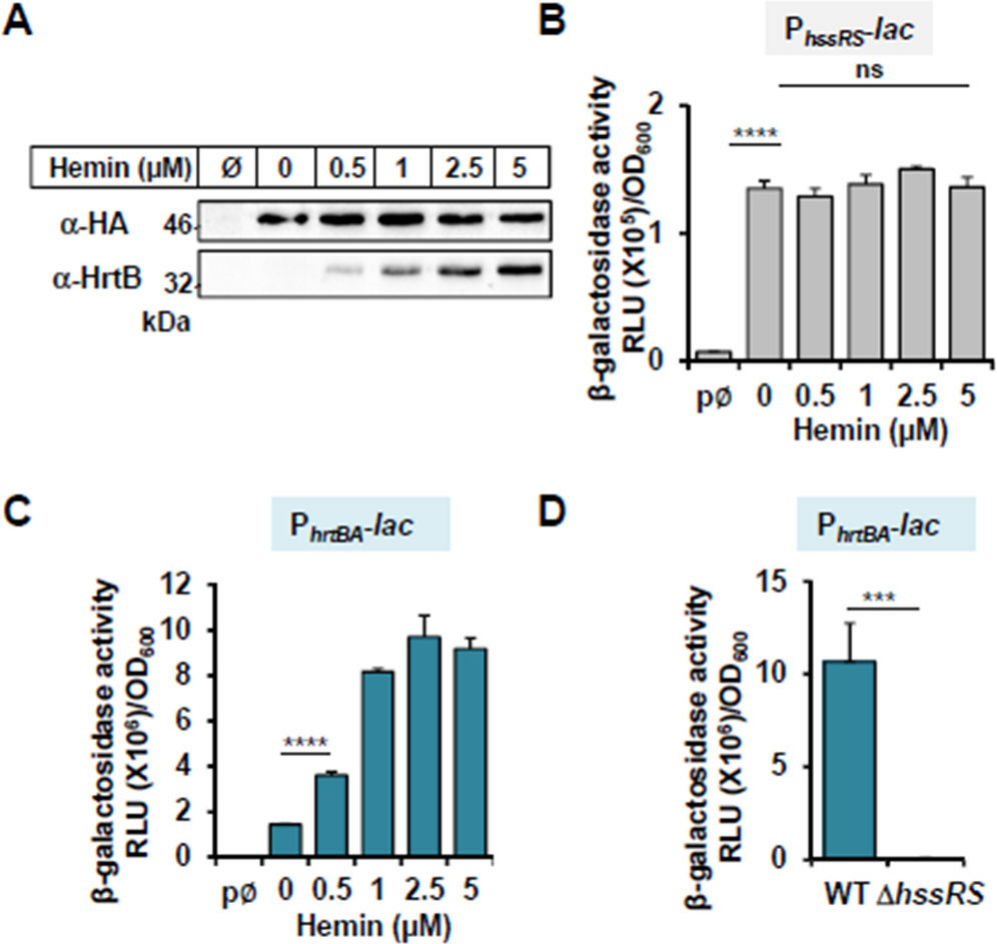
P*_hrtBA_* induction reports HssS activity. (**A**) HssS and HrtB expressions in the presence of heme. *S. aureus* HG001 Δ*hssRS*(p*hssRS*-HA) was incubated with the indicated concentrations of hemin (Ø, no hemin added). Western blot (WB) on bacterial lysates was performed with anti-HA and anti-HrtB antibodies. The result is representative of three independent experiments. (**B, C**) *hssRS* and *hrtBA* transcription regulation by exogenous heme. *S. aureus* HG001 transformed with pP*_hssRS_-lac* (**B**), pP*_hrtBA_-lac* (**C**), or pØ (promoterless pTCV-*lac*) (**B, C**) were grown in BHI to OD_600_ = 0.5 prior addition of the indicated concentration of hemin for 1.5 h. β-gal activity was quantified by luminescence. Results represent the average ± S.D. from triplicate independent experiments. ****, *P*  < 0.0001; ns, non-significant, Student’s *t* test. (**D**) P*_hrtBA_* is not induced in the HG001 Δ*hssRS* mutant. Wild-type (WT) and Δ*hssRS* HG001 strains transformed with pP*_hrtBA_-lac* were grown, and β-gal activity was determined as in panels **B and C** with 1 µM hemin. Results represent the average ± S.D. from triplicate independent experiments. ***, *P*  < 0.001, Student’s *t* test.

Heme conditions leading to the expressions of *hrtBA* and *hssS* were assessed. For this, an *S. aureus* HG001 Δ*hssRS* mutant was transformed with plasmid p*hssRS-*HA, encoding HssR and a C-terminal HA-tagged version of HssS (Table S1A). Antibodies against HA and HrtB were used for detection (Fig. 1A). Amounts of HrtB were increased in the presence of heme, as reported in *S. aureus* (13), whereas HssS expression remained constant (Fig. 1A). Accordingly, *hssRS* promoter activity, measured by β-gal expression from a P*_hssRS_-lac* fusion (pP*_hssRS_-lac*, Table S1A), was independent of heme concentration (Fig. 1B). In contrast, the P*_hrtBA_* reporter (pP*_hrtBA_-lac;* Table S1A) responded linearly with increasing concentrations of exogenously supplied hemin (Fig. 1C). As P*_hrtBA_* was specifically activated by fully induced HssRS TCS (Fig. 1D), these data establish P*_hrtBA_* induction as a specific reporter of HssRS heme sensing and signaling.

### HssS transient activation by hemin signals intracellular heme accumulation

**Fig 2:**
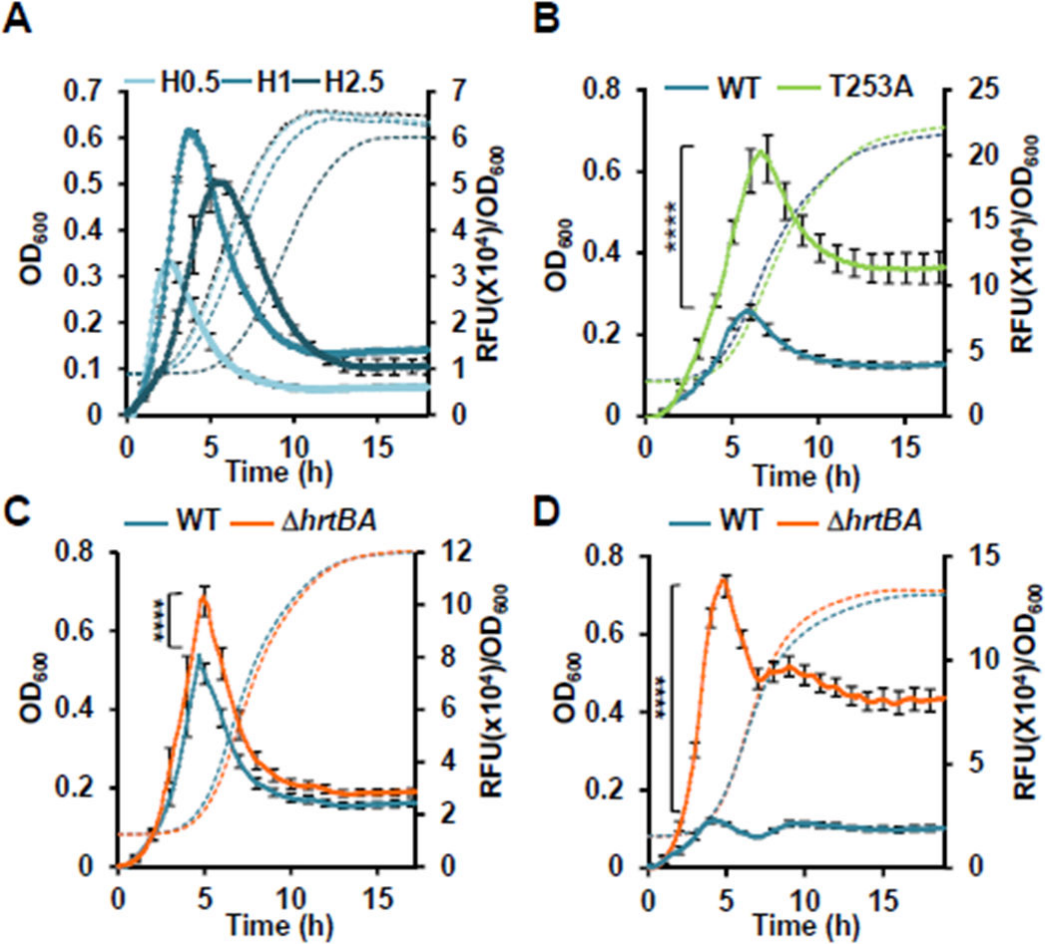
HssS transient activation reports intracellular accumulation of exogenous heme. (**A**) Dynamics of P*_hrtBA_* induction in *S. aureus* HG001 by exogenous heme. Bacteria transformed with pP*_hrtBA_*-GFP were diluted from an overnight (ON) culture to OD_600_ = 0.01 in chemical defined medium (CDM) with the indicated concentration of hemin in a microplate spectrofluorometer Infinite (Tecan). Both fluorescence (Exc: 475 nm; Em: 520 nm) and OD_600_ were recorded every 5 min for the indicated time. Fluorescence (RFU) at each time point was divided by the corresponding OD_600_. Results of hemin-induced fluorescence minus non-induced (background, 0 µM hemin) are displayed. Results represent the average ± S.D. from triplicate biological samples. The corresponding growth curves are shown. (**B**) Effect of HssS phosphatase activity. Δ*hssRS* HG001 transformed with pGFP(HssS) or pGFP(HssS T253A) were diluted in CDM ± 1 µM hemin from an ON culture in 96-microplate as in panel **A**. Fluorescence emission was quantified as in panel **A**. Fluorescence (relative fluorescence units,[RFU]) at each time point was divided by the corresponding OD_600_. Results of hemin-induced fluorescence minus non-induced (background, 0 µM hemin) are displayed. Results represent the average ± S.D. from triplicate biological samples. ****, *P* < 0.0001, Student’s *t* test. The corresponding growth curves are shown. (**C**) Dynamics of P*_hrtBA_* induction in WT and Δ*hrtBA* HG001 strains by exogenous heme. *S. aureus* HG001 WT and Δ*hrtBA* strains transformed with pP*_hrtBA_*-GFP were diluted from an ON culture to OD_600_ = 0.01 in CDM with 1 µM hemin in a 96-well microplate. OD_600_ and GFP expression were followed in a spectrofluorimeter infinite (Tecan) as in Fig. 1. Results of hemin-induced fluorescence minus non-induced (background, 0 µM hemin) are displayed. Results represent the average ± S.D. from triplicate biological samples. The corresponding growth curve is shown. ****, *P* < 0.0001, Student’s *t* test. (**D**) Dynamics of HssS activation in HG001 WT and Δ*hrtBA* strains by hemoglobin. Fluorescence emission kinetic was followed as in panel **C** in HG001 WT and Δ*hrtBA* strains transformed pP*_hrtBA_*-GFP with 0.25 µM human hemoglobin (equivalent to 1 µM hemin) added to the culture medium. Results represent the average ± S.D. from triplicate technical samples and are representative of three independent experiments. ****, *P*  < 0.0001, Student’s *t* test. The corresponding growth curves are shown.

To get insights into the mechanism of heme sensing by HssS, we followed the kinetics of HssS stimulation by heme in WT HG001 with the fluorescent reporter (pP*_hrtBA_*-GFP) (Table S1A). Hemin addition led to a transient P*_hrtBA_* response at the beginning of HG001 growth, with a maximal response output within a few hours post-heme addition (Fig. 2A). No fluorescence was detected in the strain carrying the promoterless plasmid (data not shown). At toxic heme concentrations, P*_hrtBA_* induction kinetics seems to follow the growth delay and reaches highest induction in the presence of 1 and 2.5 µM heme (Fig. 2A). P*_hrtBA_* induction phase was followed by a marked drop in fluorescence, likely corresponding to termination of the P*_hrtBA_* induction phase (Fig. 2A). In stationary phase bacteria, fluorescence associated to pP*_hrtBA_*-GFP expression stabilized following induction by heme, confirming the transient activation by HssS (Fig. S1A). Expression kinetics of GFP and HrtB correlated as shown on WB (Fig. S1B).

Negative control of HisKA is provided by its phosphatase activity on the phosphorylated regulator. The conserved catalytic histidine residue together with the adjacent conserved threonine residue (HXXXT motif) plays key roles in catalysis of phosphate hydrolysis by HisKA phosphatases (22, 23). To evaluate the role of HssS phosphatase activity in its activation dynamics, the threonine T253 was replaced by alanine in the plasmid p*hssRS*-HA, P*_hrtBA_-gfp* (pGFP(HssS)) to generate pGFP(HssS T253A) (Table S1A). HG001 Δ*hssRS* was then transformed with both plasmids, and response to heme was characterized. Higher fluorescence emission was observed in the strain expressing the HssS T253A allele compared with the WT strain but remained transient (Fig. 2B). This result illustrates the duality of HssS as a kinase and phosphatase at any time point in response to its activation by heme to finely tune the transcriptional activation of *hrtBA* by HssR.

To test the possibility that transient HssS activation was related to HrtBA-mediated heme efflux, kinetics of GFP expression from P*_hrtBA_* in Δ*hrtBA* and WT strains by subtoxic heme concentrations were compared (Fig. 2C). GFP expression was also transient in the Δ*hrtBA* strain, indicating that HrtBA expression did not explain transient HssS activation. Although P*_hrtBA_* dynamics were similar in both strains, fluorescence emission reached higher values in Δ*hrtBA* (Fig. 2C). We hypothesize that HssS activation intensity is correlated to the intracellular accumulation of heme upon *hrtBA* deletion. To test this, heme accumulation was visualized by the color of culture pellets from the Δ*hrtBA* mutant compared with the WT strain exposed to hemin (Fig. S2A). Accordingly, the Δ*hrtBA* mutant accumulated about twice more heme than did the WT as evaluated by the pyridine hemochrome assay (Fig. S2B). These results show that intracellular heme pools impact HssS activation, raising the question of where the heme-HssS interface is localized.

Replacing free hemin with Hb led to a fluorescence emission intensity that was more than five times higher in the Δ*hrtBA* strain than in the WT strain (Fig. 2D). Interestingly, the kinetics of GFP expression were modified in the presence of Hb compared with hemin. Slow and continuous delivery of hemin from Hb compared with a fast and short delivery of free hemin to the bacteria could provide an explanation for the observed distinct kinetics of GFP expression and transient HssS activation. These results further correlate membrane HssS activation with intracellular heme accumulation.

The documented role of HssS as the signal transmitter required for HrtBA expression gives strong *in vivo* evidence that HssS activation involves the pool of *S. aureus*-associated heme rather than exclusively extracellular heme as generally considered (18–20). However, as exogenous heme is detectable extracellularly, in the membrane and in the cytoplasm (10, 24, 25), we cannot discriminate which bacterial compartment drives HssS activation.

### Heme docking on a prediction model of HssS reveals a putative heme-binding region at the interface between membrane and extracellular domains

**Fig 3:**
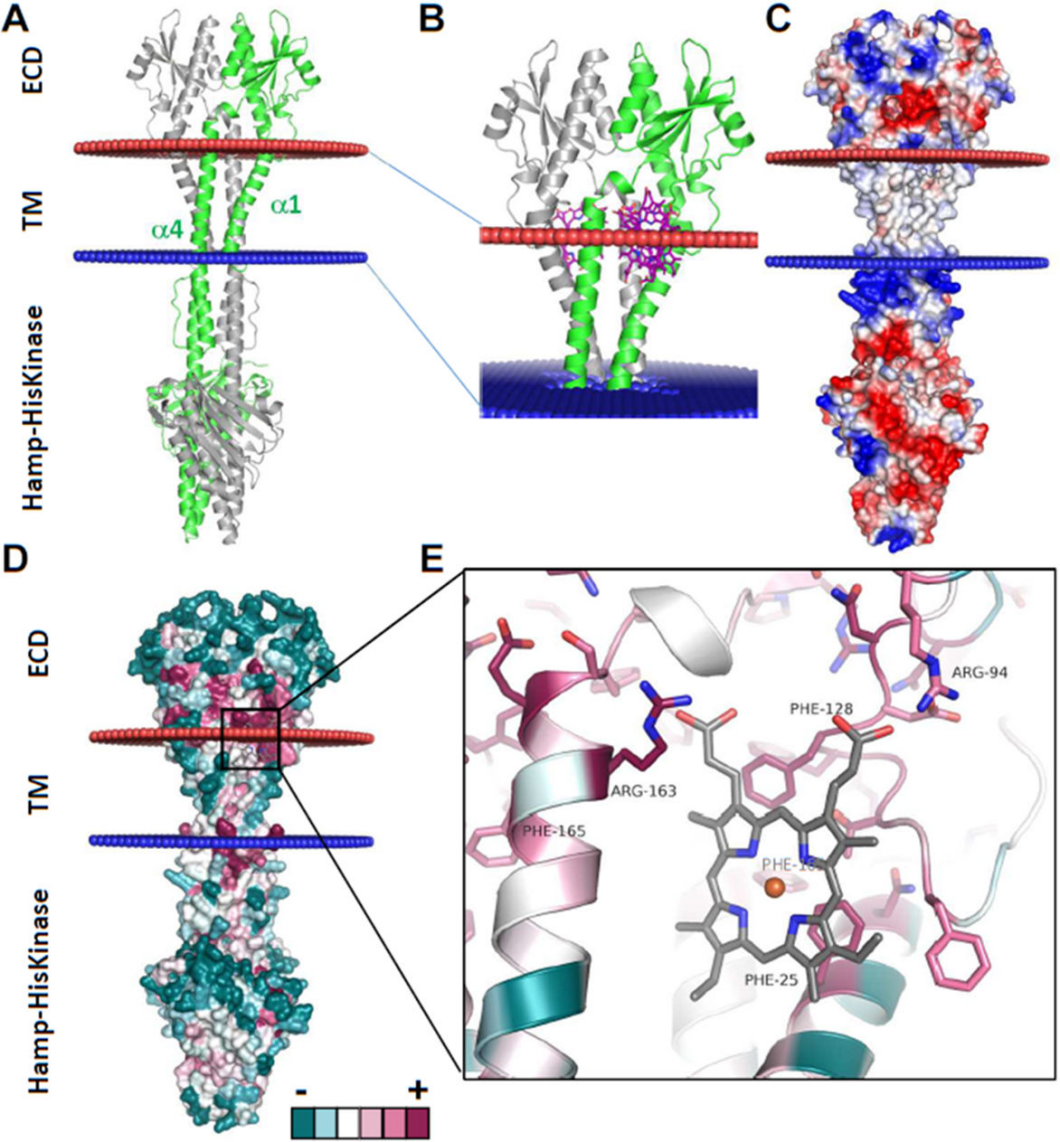
Alphafold structural model of HssS and heme docking solutions. (**A**) Alphafold model of *S. aureus* HssS dimer, with one chain colored in green and the other in gray. The position of the membrane proposed by the Orientation of Proteins in Membranes (OPM) server (https://opm.phar.umich.edu/) is shown by red and blue spheres. (**B**) Superimposition of all the docking solutions using the ECD domains of HssS. These solutions are all −8 kcal/mol and suggest the presence of two docking sites, which represent a single binding site due to the 2-fold symmetry of the dimer. This binding site is located at the interface between the membrane and the extracellular space. (**C**) Electrostatic surface potential calculated with Adaptative Poisson-Boltzmann Solver (APBS) server with a ramp from −5 kTe (red) to +5 kTe (blue). (**D**) Mapping of sequence conservation on the surface of the model depicts only a few solvent-exposed conserved residues. (**E**) Superimposition of the best docking solution of heme (colored in gray) to the model structure of HssS, with the side chains of all conserved residues within 5 Å of heme shown in a stick. Cartoon and residues are colored according to sequence conservation (Fig. 3A).

Attempts to identify specific AAs residues within HssS that may participate in heme signal reception have been hampered by the lack of an experimental three-dimensional structure. We relied on an *in silico* approach using HssS structure prediction by AlphaFold2 (AF2). Results of AF2 inferencing generated a model with a mean predicted local distance difference test (pLDDT) value of 88 (https://alphafold.ebi.ac.uk/entry/A5IVE3). This indicates a confident prediction, according to guidelines set out on EMBL’s AlphaFold Protein Structure Database, available at https://alphafold.ebi.ac.uk. Dimer prediction was obtained with Alphafold advanced (https://colab.research.google.com/github/sokrypton/ColabFold/blob/main/beta/AlphaFold2_advanced.ipynb) (Fig. 3A). The structural cytoplasmic domains of the AF2 model were consistent with previously assigned domain predictions (based on InterProScan annotations) as displayed by a prototypical HisKA (Fig. 3A). The overall structure of HssS ECD is a mixed α/β-fold with a PDC (PhoQ-DcuS-CitA)-like structure topology (26) (Fig. 3B). The central 4-stranded antiparallel β-sheet is flanked by α-helices on either side; a long N-terminal α-helix and a short C-terminal α-helix that both lie on the same side of the sheet (Fig. 3B). The long N-terminal helix is initiated by TM helix α1 (identified as residues [11-31] by Orientation of Proteins in Membranes (OPM, https://opm.phar.umich.edu/) and then participates in the mixed α/β fold of the PDC domain. A second TM helix (helix α4) (identified as residues [166-187]) allows the polypeptide chain to run in the intracellular space toward the HAMP and the HisKA domains (Fig. 3B).

We then used the program AutoDock Vina (https://ccsb.scripps.edu/) to dock heme on the surface of the HssS dimer. Docking used either the intracellular domains or the membrane and extracellular domains (Fig. 3B; Fig. S3). Using the intracellular part of HssS, all the docking solutions are above −8 kcal/mol and are scattered on the surface of the protein (Fig. S3). On the contrary, using the ECDs, all the docked solutions are below −8 kcal/mol and fall on two areas that are related by the 2-fold symmetry of the dimer, therefore representing a single binding site (Fig. 3B). This binding site located at the interface between the lipid bilayer and the extracellular space and defined by two helices α1 and α4, together with an internal loop within the ECD (Fig. 3B through D). This predicted heme-binding site is apolar on most of its surface, except for the top, which is lined with positively charged residues (Arg94 and Arg163) (Fig. 3C through E). In the best docking solution (−10.1 kcal/mol), the two heme propionates would be able to engage in a salt bridge, one with the conserved Arg94, the other with the conserved Arg163 (Fig. 3E; Fig. S4). Furthermore, heme is surrounded by a few highly conserved hydrophobic residues (Phe25, Phe128, and Phe165 belonging to the second monomer (Phe’165) being below 4 Å from the porphyrin ring (Fig. 3E; Fig. S4). This position did not reveal the usual AAs that coordinate heme such as histidine, methionine, or tyrosine. Docking of heme on *Staphylococcus epidermidis* HssS structural AF2 prediction (which shares 64% identity with *S. aureus* HssS) identified the same binding position (data not shown).

We next used a *hssS* mutational approach to challenge the proposed model of HssS heme recognition.

### Conservation of the predicted heme-binding domain is determinant for HssS activation

**Fig 4:**
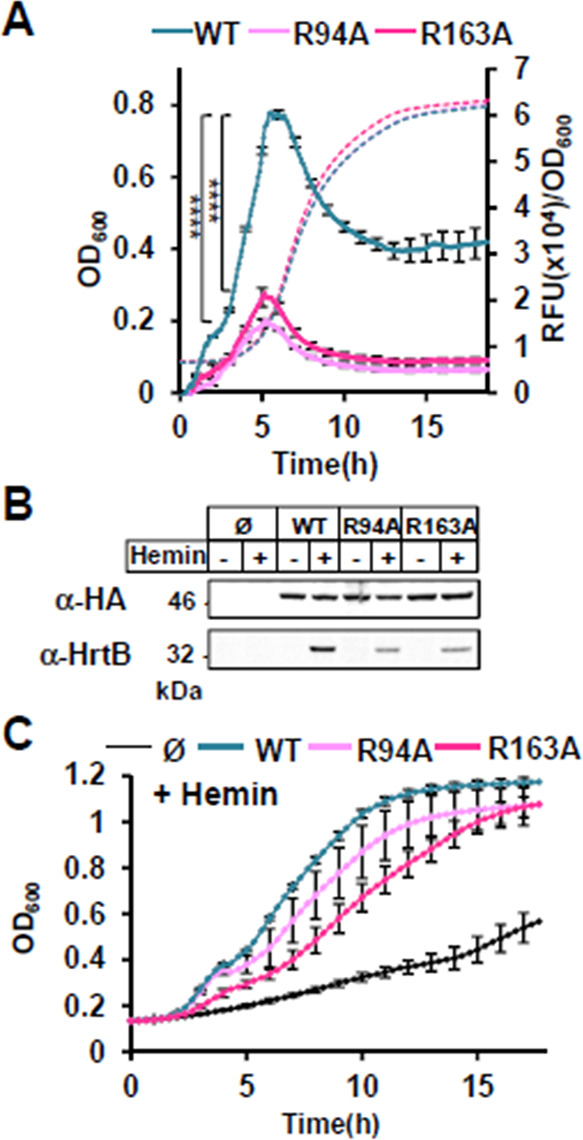
Pivotal roles of Arg94 and Arg163 on HssS activation. (**A**) P*_hrtBA_* transcriptional induction by HssS, HssS R94A, and HssS R163A. Kinetics of P*_hrtBA_* induction in HG001 Δ*hssRS* mutant transformed either with pGFP(HssS), pGFP(HssS R94A), or pGFP(HssS R163A). Strains from ON cultures were diluted to OD_600_ = 0.01 in CDM ± 1 µM of hemin in a 96-well microplate. OD_600_ and GFP expression were followed in a spectrofluorimeter infinite (Tecan) as in Fig. 2. Results of hemin-induced fluorescence minus non-induced (background, 0 µM hemin) are displayed. Results represent the average ± S.D. from triplicate biological samples. ****, *P* < 0.0001, Student’s *t* test. The corresponding growth curves are shown. Note that growth curves of WT and R94A strains overlap. (**B**) Comparative expressions of HssS-HA, HssS-HA R94A, HssS-HA R163A, and HrtB. Strains as in panel **A** were diluted in BHI from ON culture at OD_600_ = 0.01. Cultures were supplemented ± 1 µM hemin and grown for 1.5 h. HG001 Δ*hssRS* transformed with the empty plasmid (Ø) was used as a control. HssS-HA and HrtB expressions were monitored on bacterial lysates by WB with an antihemagglutinin antibody (α-HA) and an anti-HrtB antibody, respectively (α-HrtB). Results are representative of three independent experiments. (**C**) Hemin toxicity in HssS-, HssS R94A-, and HssS R163A-expressing strains. Strains as in panel **B** were diluted from an ON preculture to an OD_600_ of 0.01 in BHI supplemented with 20 µM hemin and grown in a 96 microplate. (Growth curves were similar for all strains grown without hemin (Fig. S5A.)) OD_600_ was recorded every 20 min for the indicated time in a spectrophotometer (Spark, Tecan). Results represent the average ± S.D from triplicate biological samples.

We first examined the importance of the two conserved arginines Arg94 and Arg163 in heme docking to HssS by generating alanine substitutions in pGFP(HssS) (Table S1A). The three constructs pGFP(HssS), pGFP(HssS R94A), and pGFP(HssS R163A) (Table S1A) were established in the HG001 Δ*hssRS* mutant. Activation by heme of either HssS R94A or R163A was strongly diminished compared with the WT histidine kinase as shown by the diminished fluorescence kinetic response (Fig. 4A). Although expression levels of HssS R94A, HssS R163A, and WT HssS were similar, expression of HrtB was strongly decreased in HssS point mutants (Fig. 4B). Accordingly, the two arginine HssS variant strains showed marked heme sensitivity compared with native HssS-containing strain (Fig. 4C; Fig. S5A). We conclude that HrtBA expression is strongly dependent on Arg94 and Arg163, giving support to their role in anchoring heme as predicted by docking (Fig. 4).

**Fig 5:**
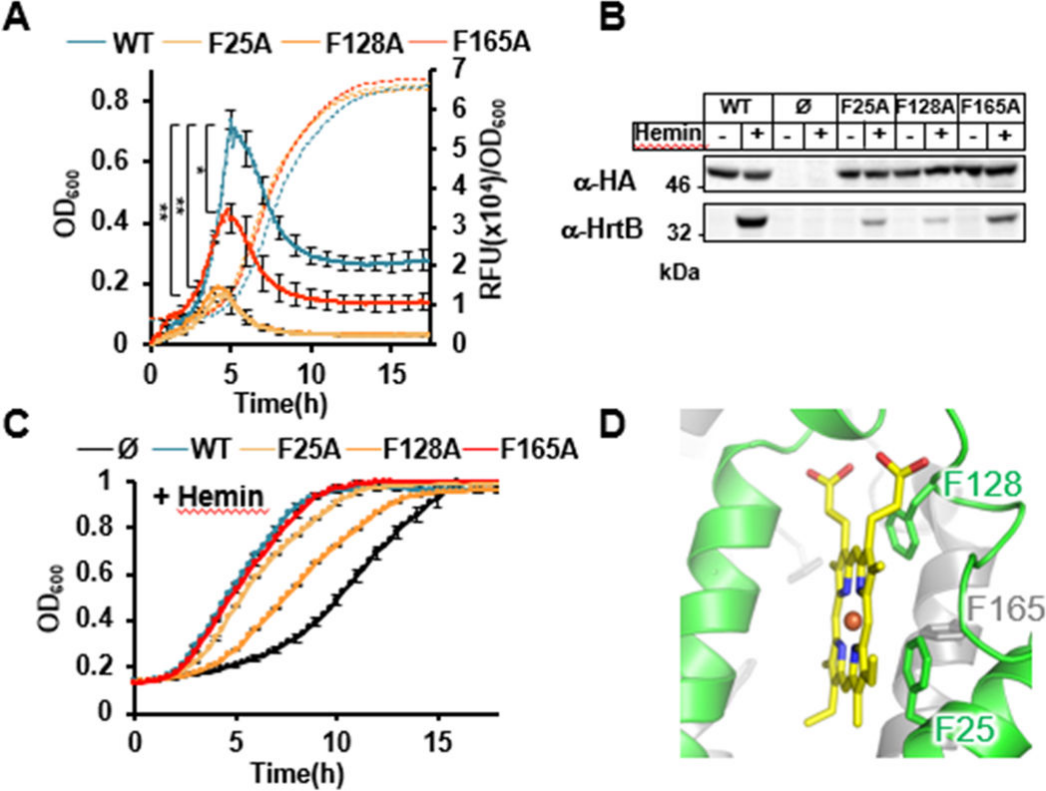
Impact of the heme-binding hydrophobic environment on HssS activation. (**A**) P*_hrtBA_* transcriptional induction by HssS, HssS F25A, HssS F128A, and HssS F165A. Kinetics of P*_hrtBA_* induction in HG001 Δ*hssRS* mutant transformed either with pGFP(HssS), pGFP(HssS R25A), pGFP(HssS F128A), or pGFP(HssS F165A) was performed as in Fig. 4. Results of hemin-induced fluorescence minus non-induced (background, 0 µM hemin) are displayed. Results represent the average ± S.D. from triplicate biological samples. ****, *P* < 0.0001, Student’s *t* test. The corresponding growth curves are shown. (**B**) Comparative expressions of HssS-HA, HssS-HA F25A, HssS-HA F128A, HssS-HA F165A, and HrtB. Strains as in panel **A** were diluted in BHI from ON culture at OD_600_ = 0.01. Cultures were supplemented ± 1 µM hemin and grown for 1.5 h. HG001 Δ*hssRS* transformed with the empty plasmid (Ø) was used as a control. HssS-HA and HrtB expression were monitored on bacterial lysates by WB as in Fig. 4. Results are representative of three independent experiments. (**C**) Hemin toxicity in HssS-, HssS F25A-, HssS F128A-, and HssS F165A-expressing strains. Strains as in panel **B** were diluted from an ON preculture to an OD_600_ of 0.01 in BHI supplemented with 20 µM hemin and grown in a 96 microplate as in Fig. 4. (Growth curves were similar for all strains grown without hemin [Fig. S5B]). Results represent the average ± S.D from triplicate biological samples. (**D**) Pymol representation of the relative positions of F25, F128, and F165 to hemin in the predicted heme-binding domain of HssS.

We next tested the importance of the predicted hydrophobic environment of heme. We choose phenylalanines, Phe25 and Phe128, which are predicted to be less than 4 Å from heme and could be engaged in π-π interactions that stabilize heme (Fig. 3E). The HG001 Δ*hssRS* mutant carrying either the F25A or F128A HssS variant (pGFP(HssS F25A) or pGFP(HssS F128A)) (Table S1A) showed similar expression levels as the WT counterpart; however, both variants were defective for heme signal transmission to P*_hrtBA_* (Fig. 5A and B). Moreover, both variants showed increased heme sensitivity (Fig. 5C Fig. S5B). As per predictions, Phe165, a conserved AA that is more distant from heme (Fig. 5D) and would only be able to contribute to heme stabilization from the edge of its aromatic ring, had a lower impact on HssS activation (Fig. 5D). We conclude that analogous to Arg94 and Arg163, Phe25 and Phe128 are important for HrtBA expression signaling and thus seem required for HssS function.

**Fig 6:**
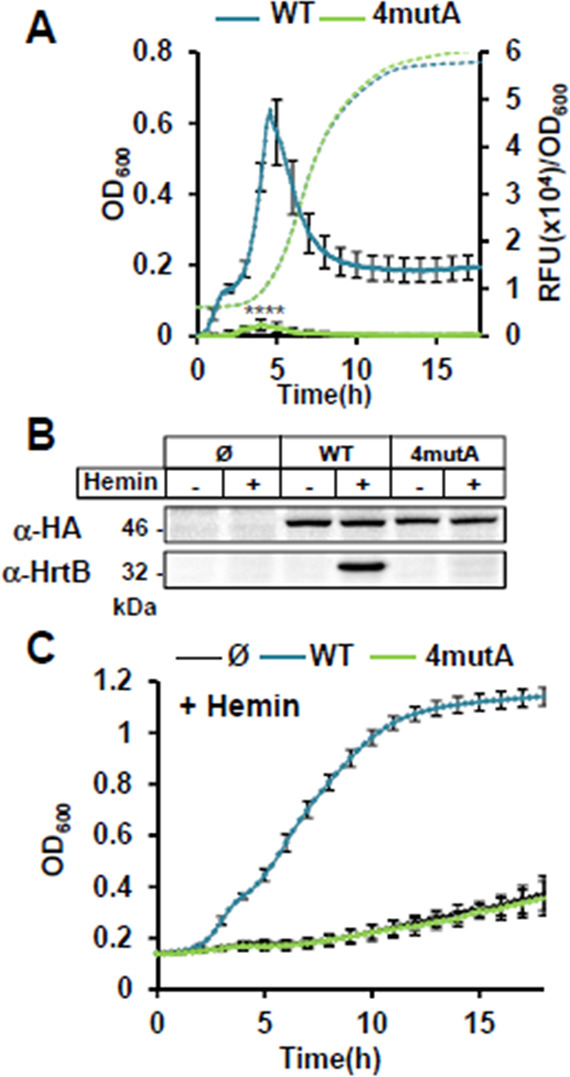
Inhibition of the heme-sensing activity of HssS R94A, R163A, F25A, and F128A (4mutA). (**A**) HssS 4mutA is unable to induce P*_hrtBA_* transcription. Kinetics of P*_hrtBA_* induction in HG001 Δ*hssRS* mutant transformed either with pGFP(HssS) or pGFP(HssS 4mutA) was performed as in Fig. 4. Results of hemin-induced fluorescence minus non-induced (background, 0 µM hemin) are displayed. Results represent the average ± S.D. from triplicate biological samples. ****, *P* < 0.0001, Student’s *t* test. The corresponding growth curves are shown. (**B**) Comparative expressions of HssS-HA, HssS-HA 4mutA, and HrtB. Strains as in panel **A** were diluted in BHI from ON culture at OD_600_ = 0.01. Cultures were supplemented ± 1 µM hemin and grown for 1.5 h. HG001 Δ*hssRS* transformed with the empty plasmid (Ø) was used as a control. HssS-HA and HrtB expressions were monitored on bacterial lysates by WB as in Fig. 4. Results are representative of three independent experiments. (**C**) Hemin toxicity in HssS and HssS 4mutA expressing strains. Strains as in panel **B** were diluted from an ON preculture to an OD_600_ of 0.01 in BHI supplemented with 20 µM hemin and grown in a 96 microplate as in Fig. 4. (Growth curves were similar for all strains grown without hemin (Fig. S5C.) Results represent the average ± S.D from triplicate biological samples.

Finally, we constructed an HssS variant with the four mutations Arg94, Arg163, Phe25, and Phe128 (pGFP(HssS 4mutA)) (Table S1A), each of which is positioned at less than 4 Å from the porphyrin (Fig. 3E). Despite being expressed at WT levels, this variant was inactive and failed to induce HrtB expression (Fig. 6A and B). As expected, heme sensitivity of the strain expressing HssS 4mutA was similar to that of the Δ*hssRS* strain in the presence of hemin (Fig. 6C; Fig. S5C). Finally, replacement of the selected AAs with the negatively charged Glu led to the same conclusions, limiting the possibility that the alanine replacements cause indirect effects on HssS, leading to a loss of its activity (Fig. S6).

Since the HssS variants tested are stable as shown by their expression on WB, our results suggest that the predicted heme anchoring AAs and the integrity of the surrounding hydrophobic environment are essential for triggering HssS activation.

### The predicted heme-binding domain mediates heme interaction with HssS

**Fig 7:**
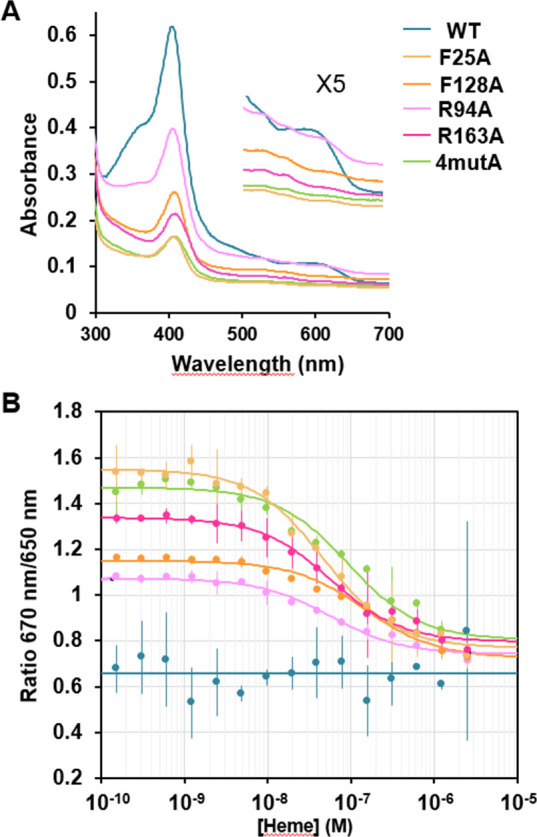
The predicted heme-binding pocket mediates heme affinity to HssS. (**A**) Comparative UV-visible absorption spectra of 15 µM HssS-His_6_ and R94A, R163A, F25A, F128A, and 4mutA variants purified on nickel affinity resin from *E. coli* membranes solubilized in DDM. inset: magnification of the 500–700 nm region. The result is representative of three independent experiments. (**B**) HssS and variants interaction with heme. The spectral shift fluorescence response curves of heme to both WT and variants reveal a heme-bound state for the WT and high-affinity heme binding sites for the variants. Data shown are representative of three independent experiments, and the error is calculated as standard deviation.

To further discard the possibility that our mutational analysis led to allosteric modifications of the structure and/or dynamics of HssS without interfering with heme binding, HssS and its variants (F25A, F128A, R94A, R163A, and 4mutA) tagged at their Ct with 6XHis were purified in parallel from *Escherichia coli* membrane fraction using n-dodecyl-β-D-maltoside (DDM). All proteins were isolated at similar concentrations, suggesting that they were correctly folded and targeted to *E. coli* membrane (Fig. S7). UV-visible absorption spectroscopy of HssS WT (Fig. 7A) exhibited a sharp Soret peak at 407 nm indicating unambiguously that the protein copurified with heme *b* from *E. coli* as previously observed for bacterial hemoproteins (10, 12). In contrast, HssS variants copurified with lower and various proportions of heme as seen by the intensity of their Soret peaks, suggesting a loss of their affinity of heme compared with the WT (Fig. 7A). A shift in the Soret peak from 407 to 409 nm, as well as a distinct profile in the 500–700 nm region, suggests that the environment of heme that still bind to the variants slightly differs from WT.

Finally, to determine whether the four residues mutated in the 4mutA variant were involved in heme binding, we designed a ratiometric fluorescence assay (Fig. 7B). Since WT HssS co-purified with heme (Fig. 7A), we expected little or no change along the titration, depending on the degree of heme saturation. Indeed, the 650 nm/670 nm ratio was stable at around 0.65 during the titration, indicating no heme binding to the protein, probably because HssS was already fully saturated (Fig. 7B). Since the four mutA variant was purified bound to significantly less heme than the WT, we expected to measure an affinity that would result from binding to the apo-protein form. Indeed, the ratiometric fluorescence assay showed a single wave of binding with a 650 nm/670 nm ratio that stabilized at around 0.6 at high heme concentration and increased to 1.5 at low heme concentration (Fig. 7B). The fit of this wave gives a *K*_d_ of 0.91 µM, which should be taken as an approximation, since the protein concentration corresponds to a mixture of apo- and holo-proteins. The four individual mutants were also co-purified with various proportions of heme, and we were able to determine their relative affinity with heme, which varies from 0.4 to 1.4 µM (R163A: 0.55 µM; R93A: 0.54 µM; F25A: 0.46 µM; and F128A: 1.45 µM). Therefore, all the mutations we tested contribute to heme stabilization, although they do not seem to have a clear additive effect. Nevertheless, the fact that the 650 nm/670 nm ratio is stabilized at a value of around 0.6 at high heme concentration for both the WT and all the variants suggests that the WT is stabilized in a heme-bound conformation, consistent with its co-purification with heme. This in turn suggests that the affinity of the WT protein for heme is in the low pM range, as there is no detectable dissociation at a heme concentration of 750 pM (the lowest heme concentration tested in the WT titration).

### HssS lacking the extracellular domain [42-151] is constitutively activated

Our findings question the role and importance of extracellular domain (ECD) of HssS. We examined the impact of removing most of the [35-168] domains of HssS corresponding to the ECD on heme signal transduction. A truncated version of *hssS* was constructed (referred to as pGFP(HssS ΔECD)) (Table S1A) and was established in HG001 Δ*hssRS*. In this variant, the ECD AAs comprising AAs [35-41] and [151-168] were conserved and fused to allow membrane insertion and thus lacked Arg94 and Phe128, which are important for heme response (see above). Expression and membrane localization of HssS-HA ΔECD were verified on WB using an anti-HA antibody following cell fractionation (Fig. S8A). Expression of the ECD variant compared with the full-length protein was lower, possibly suggesting differences in protein stability (Fig. S8A).

**Fig 8:**
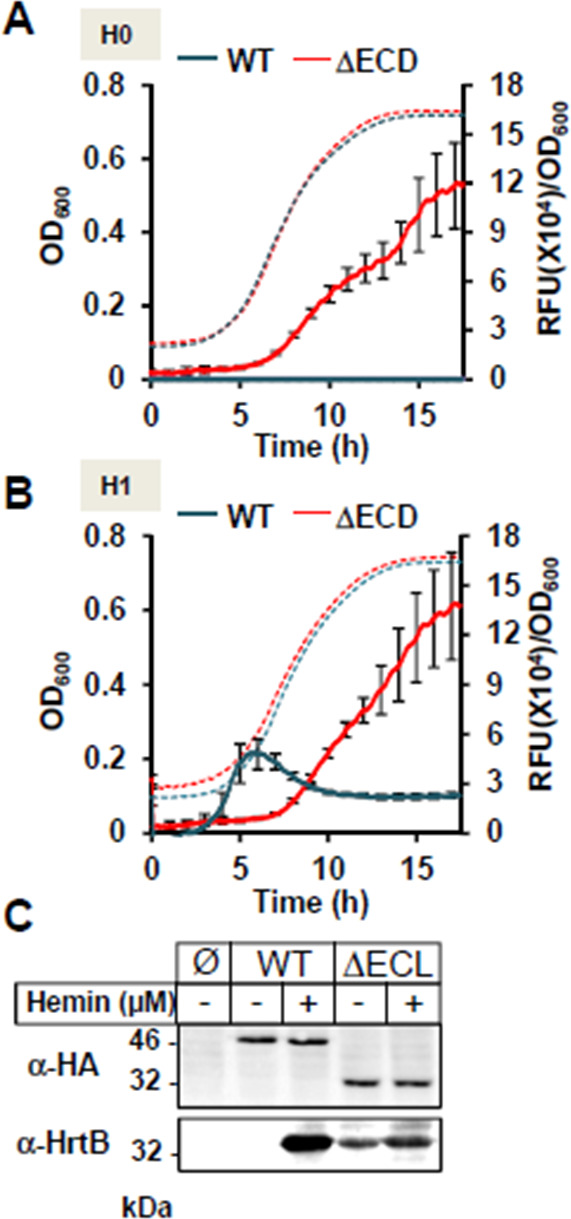
The extracellular domain is required for heme-dependent activation of HssS. (**A-B**) Comparative dynamics of P*_hrtBA_* transcriptional induction by HssS WT and HssS ΔECD HG001 in the absence (**A**) or presence of hemin (**B**). HG001 Δ*hssRS* mutants transformed with either pGFP(HssS), pGFP(HssS ΔECD), or empty vector were grown as in Fig. 2 in CDM without heme (**A**) or supplemented with 1 µM of hemin (**B**). Fluorescence (RFU) and OD_600_ were determined as in Fig. 2. Results of fluorescence (RFU/OD_600_) from strains expressing HssS WT or HssS ΔECD minus empty vector transformed strain are displayed. Results represent the average ± S.D. from triplicate biological samples. The corresponding growth curves are shown. (**C**) HssS ΔECD constitutively signals HrtB expression. Δ*hssRS* HG001 transformed either with pGFP(HssS), pGFP(HssS ΔECD), or empty vector (Ø) were used to monitor HssS and HrtB expressions by WB with α-HA and an α-HrtB, respectively. Bacteria were grown to an OD_600_ = 0.5 and induced for 1.5 h ± 1 µM hemin. SDS-PAGE was performed on cell lysates (25  µg per lane). Results are representative of three independent experiments.

To investigate the impact of the ECD deletion on HssS activity, GFP expression from pGFP(HssS) and pGFP(HssS ΔECD) was followed in the absence or presence of 1 µM heme (Fig. 8A and B). Remarkably, P*_hrtBA_*-GFP was expressed constitutively and independently of heme addition in the strain carrying pHssS ΔECD (Fig. 8A and B). However, although HrtB expression was constitutive, its levels were lower than in the strain-producing WT HssS (Fig. 8C; Fig. S8A). Interestingly, despite lower levels of HrtB in the strain expressing HssS ΔECD, the strain showed markedly improved fitness when challenged with 10 µM heme when compared with the isogenic strain expression HssS WT (Fig. S8B and C).

We conclude that in the absence of ECD, HssS does not retain its capacity to be stimulated by heme but also appears to transmit a basal signal, leading to *hrtBA* induction and bacterial protection against heme.

## DISCUSSION

Heme homeostasis in Gram-positive bacteria is mainly achieved *via* heme efflux. In *S. aureus*, the major heme efflux transporter HrtBA is controlled by HssRS (13, 18, 27). Our results give strong support that the heme-sensing function of the dimeric HK HssS is located in a structural domain at the interface between the TM and extracellular domains. Thus, membrane-attached rather than extracellular heme pools control HssS transient activation, shedding new light on a detoxification mechanism of an abundant host molecule in a major human pathogen, *S. aureus*.

Five lines of evidence support the hydrophobic HssS interface as being required for heme binding. (i) In-depth *in silico* modeling predicted HssS protein structure and heme-binding candidate amino acids. In heme docking simulations, heme interacted with both periplasmic and lipid-embedded amino acids. (ii) Loss of functional HssS activity correlated with directed substitutions of heme-binding candidate amino acids, consistent with their functional roles. Replacement of targeted AAs in the hydrophobic environment and the two predicted anchoring arginines (Arg94 and Arg163) were all required to abolish HssS activation. (iii) The same AAs substitutions led to a severe decrease in the affinity of heme for the HK, providing direct evidence that heme binding to the predicted domain correlates to HssS activation. (iv) Involved amino acids and overall structure of *S. aureus* HssS are conserved in protein homologs in other bacteria. These findings also clarify previous work in which conserved substitutions of conserved AA residues required for HssS heme sensing in *S. aureus* and *B. anthracis* were mapped to the same domain (19, 20). (v) Heme availability from the outside or from the inside led to similar kinetics of induction. HssS is activated not only by extracellular heme but also by increasing intracellular heme pools (e.g., in a Δ*hrtBA* mutant), suggesting that both heme sources are accessible to HssS binding; by deduction, this common site would need to be the membrane.

Our findings implicate heme bound to the HssS membrane-outer surface interface as an activation signal of HssS. We suggest that this mechanism of TCS activation may more generally be a novel basis for hydrophobic molecule sensing. A previously reported class of HKs called intramembrane histidine kinase (IM-HK) perceives its stimuli in the membrane, but not directly (28–30). Instead, an N-terminal signal transfer region consisting of two transmembrane helices presumably connects the IM-HKs with the regulated accessory membrane proteins that function as the true sensors. For HKs that lack most of the sensory domain in the ECD, cell envelope stress sensing has been directly linked to the ABC transporter via TM-TM interactions (28–30). In contrast, an activation mechanism based on direct physical interaction between HssS and HrtBA seems unlikely since P*_hrtBA_* is induced in a *hrtBA* deletion mutant. HssS is thus not activated similarly to IM-HK. We thus propose HssS as a paradigm for signaling by organic molecules, as produced by the host, which are in contact with the membrane-surface interface. Membrane inputs could be particularly relevant for regulators of efflux transporters controlling exogenous substrates, including antibiotics (31–33).

*In silico* simulation revealed the membrane-surface interface as the site of HssS interaction with heme but does not take into account the role of the flexible phospholipid environment, which varies according to conditions and environmental lipids (34). Since intramembrane heme concentrations impact HssS activation (as seen by testing HssS induction in the Δ*hrtBA* mutant), it is highly likely that heme crosses the membrane to activate HssS. Interestingly, the structures of two membrane heme-binding proteins, HrtB (the permease regulated by HssS) and CydDC, have been solved (17, 35). Similar HssS, their heme-binding domains are located within a cavity formed by transmembrane helices beneath the ECD in the vicinity of the membrane plane. The two heme propionates groups form electrostatic interactions with two arginines in CydDC (35). Our model of heme interaction with HssS is therefore in line with these recent findings.

**Fig 9:**
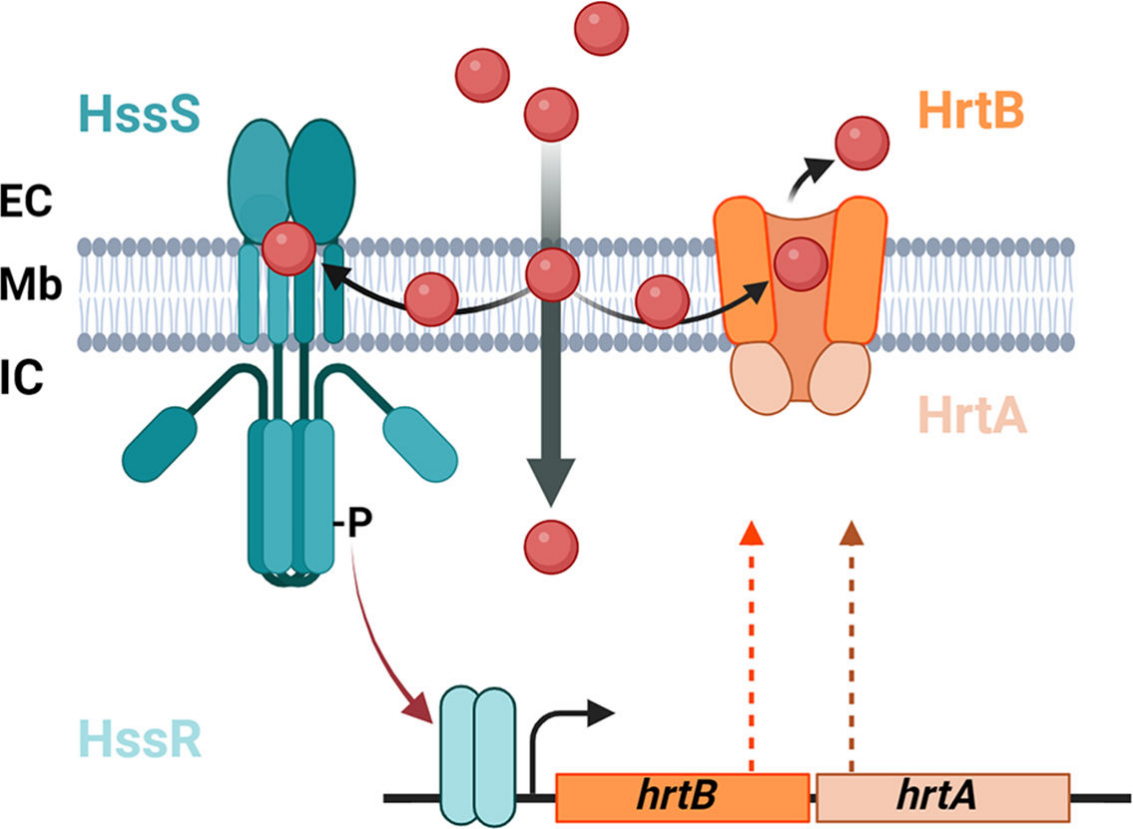
Functional model of exogenous heme management by the gatekeeping HssRS-HrtBA system in *S. aureus.* Exogenous hemin (red dots) translocates through the membrane (Mb) compartment from the extracellular (EC) to the intracellular (IC) compartments. HssS senses heme at the membrane interface, activating the phosphorelay between the HK and HssR leading to the expression of HrtBA. The pool of hemin^3^ that crosses the membrane by diffusion is counterbalanced by HrtBA that extrudes heme from the membrane to EC space (17).

Our findings that membrane heme activates HssS provide a functional link between HssS and HrtBA. HrtB is a member of the MacB family of efflux pumps that is distinct from other structurally characterized ABC transporters (36). Heme efflux by HrtB is initiated by a heme-binding site in the outer leaflet of the membrane, which is laterally accessible to heme (17). HssS would have the integral role as heme “gatekeeper” that controls exogenous heme pools to prevent translocation within the membrane and into the cytosol (Fig. 9). Membrane heme may either enter passively into the intracellular compartment or be effluxed by HrtB before this step. This alternative model is compatible with our observations that exogenous excess heme is internalized in *S. aureus* independently of the Isd heme import system in our experimental conditions ((37) and data not shown).

Unlike *S. aureus*, *L. lactis* and *E. faecalis* control HrtBA expression using intracytoplasmic TetR transcriptional regulators (HrtR in *L. lactis* (12) and FhtR in *E. faecalis* (10)). It is thus tempting to speculate that the HssS-sensing mechanism discriminates between heme originating from endogenous synthesis and exogenous sources, which would minimize interference with metabolic processes. *S. aureus* is a serious threat to public health due to the rise of antibiotic resistance in this organism. As such, many efforts are underway to develop therapies that target essential host adaptive processes in *S. aureus*. Our findings provide a new basis for the elucidation of pathogen-sensing mechanisms as a prerequisite to the discovery of inhibitors.

## MATERIALS AND METHODS

### Bacterial growth conditions and media

*S. aureus* HG001 and their derivatives (bacterial strains, plasmids, and mutant constructions procedures are outlined in the supplemental data section (Text S1; Table S1)) were grown as ON pre-cultures at 37°C in bovine heart infusion (BHI) liquid broth (Becton Dickinson, France) supplemented with 0.2% (wt/vol) glucose with aeration by shaking at 200 rpm. All growth assays were performed in a 96-well plate in 200 µL of BHI. Optical density at 600 nm (OD_600_) served as a measurement of growth and was measured every 15 min for the indicated total time in a microplate reader (Spark, Tecan). *E. coli* strains were grown in a lysogeny broth (LB) medium with aeration by shaking at 180 rpm. When needed, antibiotics were used as follows: 50 µg/mL kanamycin and 100 µg/mL ampicillin for *E. coli*; 5 µg/mL erythromycin for *S. aureus*. Hemin was prepared from a stock solution of 10 mM hemin chloride dissolved in 50 mM NaOH; Frontier Scientific).

### Dynamics of fluorescence emission

For kinetic studies using GFP, *S. aureus* strains were grown on a 96-well plate in 200 µL chemically defined medium (CDM) as reported (38, 39). CDM medium contained around 170 nM iron (38). CDM is uncolored, therefore minimizing fluorescence background. OD_600_ and fluorescence (expressed in relative fluorescence units, RFU) (Exc.: 480 nm; Em.: 515 nm, bandwidth: 9 nm, integration time: 40 µs, gain: 140) were measured every 5 min in a black 96-well microplate with transparent bottom (Greiner Bio-one, Kremsmünster, Austria) in a spectrofluorimeter (Infinite M200, Tecan) at 37°C under constant shaking (orbital, amplitude: 2.5).

### Immunoblotting

Bacteria were pelleted by centrifugation and washed with PBS. Resuspended cells were then pelleted at 3,500 × *g* for 10 min, resuspended in 50 mM Tris-HCl (pH 7.5), 150 mM NaCl, containing 0.2% (vol/vol) Triton (lysis buffer), and disrupted with glass beads (Fastprep; MP Biomedicals). Cell debris was removed by centrifugation at 18,000 × *g* for 15 min at 4°C. To prepare membranes, bacterial lysates were prepared as above except that bacteria were lysed in 20 mM Tris-HCl (pH 7.5). Lysates were then subjected to centrifugation at 100,000 × *g* for 45 min at 4°C in an ultracentrifuge Beckman XL-90 (Beckman France, Villepinte) equipped with a 70.1T1 rotor. Membrane pellets were resuspended in lysis buffer. Proteins were quantified by the Lowry Method (Bio-Rad) and denatured in Laemmli sample buffer at 95°C for 5 min and processed for SDS-PAGE and immunoblotting. Antibodies used were produced or purchased are outlined in Text S1.

### Heme docking analysis

Heme (https://www.rcsb.org/) was docked onto the modeled HssS structure with AutoDock Vina 1.1.2 (https://ccsb.scripps.edu/). The exhaustivity parameter was set to 8. Ligand and protein coordinates were prepared (including polar hydrogen atoms and atoms charges to take hydrogen and electrostatic interactions into account) using Open babel (http://openbabel.org). Docking of heme was performed on the entire surface of one monomer in the dimeric HssS with 100 boxes (27.10^−33^ × m^−3^). From each box, the top scoring pose (in terms of binding free energy (kcal/mol) as estimated by AutoDock Vina) was selected for the binding site. The 10 top docking solutions were visualized with Visual Molecular Dynamics (VMD) software (40; http://www.ks.uiuc.edu/Research/vmd/) and designated a single periplasmic domain.

### Purification of HssS-His_6_ and HssS 4mutA-His_6_

*E. coli* BL21 C43 (DE3) (1 L culture volume) transformed with p*hssS-his_6_*, p*hssS* F25A*-his_6_*, p*hssS* F128A*-his_6_*, p*hssS* R94A*-his_6_*, p*hssS* R163A*-his_6_,* and p*hssS* 4mutA-*his_6_* (Table S1A) were grown to OD_600_ = 0.6 at 37°C, and expression was induced with 1 mM isopropyl 1-thio-β-d-galactopyranoside (IPTG) ON at RT. Cells were pelleted at 3,500 × *g* for 10 min and then resuspended in 20 mL lysis buffer (50 mM Tris-HCl [pH 8], 300 mM NaCl, 30% glycerol) and disrupted with glass beads (Fastprep, MP Biomedicals). Cell debris was removed by centrifugation at 18,000 × *g* for 15 min at 4°C, and membranes were pelleted by ultracentrifugation at 100,000 × *g* for 2 h at 4°C in an ultracentrifuge Beckman XL-90 (Beckman France, Villepinte) equipped with a SW41T1 rotor. Purification of full-length recombinant HssS-His_6_ and HssS 4mutA-His_6_ was performed according to a modified protocol of recombinant histidine kinase purification (41). Briefly, the membrane pellet was resuspended in 4 mL lysis buffer containing 20 mM DDM and 20 mM imidazole. The resuspended membrane lysate was mixed with 1 mL nickel affinity resin (Invitrogen) and incubated on a spinning wheel at 4°C for 2 h. The lysate/resin mixture was loaded onto a polypropylene column. The lysate was run through, and the resin was washed three times with 20 mL lysis buffer containing 20 mM imidazole and 4 mM DDM. HssS-His_6_ and HssS 4mutA-His_6_ were eluted with 8 × 200 µL with lysis buffer containing 300 mM imidazole and 4 mM DDM. A 10 µL sample of each fraction was used for SDS-PAGE and Coomassie staining. The fractions containing the highest yield of the protected were pooled into a dialysis tube (D-Tube dialyzer, 3.5 kDa MWCO, Merck). The dialysis tube was incubated in 400 mL dialysis buffer (50 mM HEPES, 200 mM KCl, 50% [vol/vol] glycerol, pH 8) for 2 h at 4°C with constant stirring. The dialysis buffer was replaced twice more (2 h and 16 h). The proteins were recovered from the dialysis tube and finally stored at −80°C. The concentrations of the proteins were determined with the extinction coefficient of HssS-His_6_ (25.33 mM^−1^ cm^−1^) in a spectrophotometer (Infinite M200, Tecan).

### Ratiometric fluorescence assay

Binding was calculated for HssS WT, the four individual mutants (HssS F25A, HssS F128A, HssS R94A, HssS R163A) and the 4mutA variant using spectral shift response curves. A range of heme concentration (from 0.25 µM to 763 pM) was incubated with 20 nM (WT, F25A, and four mutA variants) or 50 nM protein (F128A, R163A, and R93A variants), which were covalently labeled with the fluorescent dye Monolith RED-NHS according to the instructions provided by the manufacturer (Nanotemper Technologies, Germany). The labeled proteins were incubated in 50 mM HEPES (pH 8.0), 200 mM KCl, 5% (vol/vol) glycerol, and 0.05% (vol/vol) Tween for 30 min at RT in the dark with equal volumes of 16 serial dilutions of heme. These reactions were then measured using the Nanotemper Monolith X, equipped with Spectral Shift and microscale thermophoresis technologies. Each assay was performed in independent triplicate, and the data were analyzed by the instrument’s software (MO control v2.5.4.).

## SUPPLEMENTAL MATERIAL

Text S1 mbio.00230-24-s0001.docx Supplemental methods and table and figure legends.

Fig. S1 mbio.00230-24-s0002.tif Transient induction of P_*hrtBA*_ during stationary and exponential growth phases.

Fig. S2 mbio.00230-24-s0003.tif Heme accumulates in HG001 Δ*hrtBA* strain.

Fig. S3 mbio.00230-24-s0004.tif Superimposition of all the docking solutions using the intracellular part of HssS.

Fig. S4 mbio.00230-24-s0005.tif WebLogo representation of AAs 1-188 of HssS.

Fig. S5 mbio.00230-24-s0006.tif Growth of HG001 ∆*hssRS* complemented with pGFP(HssS) or HssS variants.

Fig. S6 mbio.00230-24-s0007.tif Comparative effect of replacing Arg94, Arg163, Phe25, and Phe128 with Ala or Glu on HssS activation.

Fig. S7 mbio.00230-24-s0008.tif Purification of HssS and HssS variants from *E. coli*.

Fig. S8 mbio.00230-24-s0009.tif HssS ΔECD is expressed at the membrane and signals heme detoxification.

Table S1 mbio.00230-24-s0010.docx Strains, plasmids, and oligonucleotides.

ASM does not own the copyrights to Supplemental Material that may be linked to, or accessed through, an article. The authors have granted ASM a non-exclusive, world-wide license to publish the Supplemental Material files. Please contact the corresponding author directly for reuse.
