# Supplementary material for: HssS activation by membrane heme defines a paradigm for two-component system signaling in Staphylococcus aureus
Source: mBio. 2024 Apr 29;15(6):e00230-24. doi: 10.1128/mbio.00230-24 (PMC11237747; doi:10.1128/mbio.00230-24)
Supplement: Text S1 — Supplemental methods and table and figure legends. [file mbio.00230-24-s0001.docx]

**Text S1.** **Supplemental Methods, Table and Figure legends**

**Supplemental Methods**

**Bacterial strains.** The strains and plasmids used in this work are listed in [Table S1](http://www.jbc.org/cgi/content/full/M111.297531/DC1)A. *Staphylococcus aureus* strain HG001 is a derivative of the RN1 ([NCT8325](http://clinicaltrials.gov/show/NCT8325)) strain with restored *rbsU* (a positive activator of SigB) (1).

**Plasmid construction**. Plasmid pTCV-*lac* is a low copy number plasmid that uses the *lac* gene as a reporter to evaluate promoter activities in Gram-positive bacteria (2) (Table S1A). DNA fragments containing the *hrtBA* or the *hssRS* promoter were PCR-amplified with primer pairs (O1-O2) or (O3-O4) (Table S1B) respectively. The amplified fragments were digested with *Eco*RI/*Bam*HI and cloned into pTCV-*lac*, resulting in plasmids pP_hrtBA_-*lac* and pP_hssRS_-*lac* (Table S1A). pP*_hrtBA_*-GFP (Table S1B) was constructed by cloning fragments corresponding to P*_hrtBA_* with the primer pairs (O5-O6) and into the *Bam*H1/*Eco*R1 restriction sites of the pCN52 vector (Table S1A) using HG001 genomic DNA as template. The DNA sequence of P*_hssRS_ hssRS*-HA was PCR amplified from the plasmid pUC *hssRS*-HA-P_hrtBA_ with oligonucleotides (O7-O8) (Table S1B), digested with *Bam*H1/*Kpn*1 and ligated into pCN52 to give rise to p*hssRS*-HA, P*_hrtBA_*-GFP (pGFP(HssS)) (Table S1A). *hssS* gene is followed by the nt sequence encoding the hemagglutinin influenza epitope (HA, YPYDVPDYA). p*hssRS*-HA ΔECL, P*_hrtBA_*-GFP (pGFP (HssS ΔECL)) was obtained by an overlap of two PCRs amplified with the primer pairs (O7-O9) and (O8-O10) (Table S1B) using pUC *hssRS*-HA-P*_hrtBA_* as a template. The P*_hssRS_ hssRS*-HA ΔECL fragment digested with *Bam*H1/*Kpn*1 and ligated into pCN52 to give rise to pGFP (HssS ΔECL) (Table S1A)*.* The DNA sequence corresponding to extracellular domain of HrtB was PCR amplified with primers pair (O11-O12) using HG001 genomic DNA as a template that was cloned into the *E. coli* expression vector pET200 as recommended by the manufacturer’s instructions to generate pET200-*hrtB* ECL (Table S1A). The pMADΔ*hrtBA* plasmid was constructed as follows: 2 DNA fragments of ~800 bp flanking the *hrtBA* operon were PCR-amplified using *S. aureus* HG001 genomic DNA as a template and primer pairs (O13-O14) and (O15-O16) (Table S1B). Both fragments were used as templates in a second round of PCR amplification with primers (O13-O16), resulting in an overlapping ~1.6 kb fragment, which was digested by *Xma*I and *Bam*HI and cloned into the thermosensitive plasmid, pMAD (2), giving rise to pΔ*hrtBA* (Table S1A). pΔ*hrtBA* was established by transformation in *S. aureus* HG001. The double cross-over event leading to the Δ*hrtBA* mutant was obtained as described (3). p*hssRS*-HA was constructed by PCR amplification of the hssRS-HA sequence from the pUC *hssRS*-HA-P*_hrtBA_* plasmid with (O17-O18) (Table S1B). The amplified P*_hssRS_ hssRS*-HA DNA fragment was cloned into *Pst*1/*Bam*H1 restriction sites of PAW8 (Table S1A). p*hssS*-*his_6_* p*hssS-his_6_*, p*hssS* F25A*-his_6_*, p*hssS* F128A*-his_6_*, p*hssS* R94A*-his_6_*, p*hssS* R163A*-his_6_* and p*hssS* 4mutA-*his_6_* were constructed by PCR amplification of *hssS* and *hssS* mutants using the corresponding plasmids pGFP(HssS) plasmids with (O19-O20). The inserts were cloned into the *BamH*1/*Xho*1 restriction sites of pET2160 (Table S1A). All plasmids were verified by DNA sequencing.

**Antibodies.** An anti-HrtB antibody targeted to the extracellular domain of *S. aureus* HG001 HrtB was produced. The HrtB[45-236] fragment was purified from *E. coli* as a His-tagged antigen from the plasmid pET200-*hrtB* ECL (see above and Table S1A), purified from bacterial lysates on a nickel affinity resin (His-Select, Sigma-Aldrich) as described (4). Briefly, E. coli BL21 (DE3) (Thermo Fisher France, Villebon-sur-Yvette) transformed with pET200-*hrtB* ECL was grown to OD_600_ = 0.6, and expression was induced with 1 mM IPTG for 2 h at 37 °C. Cells were pelleted at 3,500 × g for 10 min, resuspended in 50 mm Tris-HCl, pH 8.0, 300 mm NaCl, containing 20 mM imidazole (binding buffer), and disrupted with glass beads (Fastprep, MP Biomedicals France, Illkirch-Graffenstaden). Cell debris were removed by centrifugation at 18,000 × g for 15 min at 4 °C. The soluble fraction (surpernatant) was mixed with nickel affinity resin (Invitrogen) and incubated on a spinning wheel at 4°C for 1 h. The resin was then centrifuged (1,300 × g, 5 min) and washed three times with binding buffer. Purified proteins were eluted with 50 mM Tris-HCl, pH 8.0, 300 mm NaCl, containing 150 mm imidazole, dialyzed against 50 mM Tris-HCl, pH 7.5, and finally stored at −80 °C. Protein concentrations were determined with the Lowry assay method (Bio-Rad France, Marnes-la-Coquette). The resulting purified His-HrtB-ECL was used for rabbit antibody production (Covalab, Bron, France). Antiserum specificity was determined by Western blots using known amounts of purified His-HrtB-ECL proteins and bacterial lysates expressing HrtB. The polyclonal anti-GAPDH antibody was a generous gift from F. Götz (University of Tübingen, Germany) (5). The polyclonal anti-HA and anti-His antibodies were purchased from Thermo Fischer.

**β-galactosidase assays.** β-galactosidase activity was quantified by luminescence in an Infinite M200 spectrolumineter (Tecan) using the luminescence β-glo assay as recommended by the manufacturer (Promega France, Charbonnières-les-Bains, France). Briefly, *S. aureus* strains cultures were diluted from ON precultures in BHI to an OD_600_ = 0.01 and grown to an OD_600_ = 0.5 and then incubated for 1 h with the indicated concentrations of hemin. 25 µl cultures were distributed in a white 96-wells microplate (Greiner Bio-one) in triplicate. 50 µl β-glo assay reagent was added per well. After 10 min incubation at RT, luminescence was quantified. In parallel, 200 µl of the corresponding cultures were distributed in a transparent 96-wells plate to measure the OD_600_  for normalization of the luminescence.

**Heme concentration determination in bacterial lysates.** Proteins from bacterial lysates (as described above) (in a volume of 250 µl) were mixed with 20 µl of 0.2 M NaOH, 40 % (v/v) pyridine and 500 µl potassium ferricyanide or 5 mM sodium dithionite. 500-600 nm absorption spectra were recorded in a UV-visible spectrophotometer Libra S22 (Biochrom, Cambridge,UK). Dithionite-reduced minus ferricyanide-oxidized spectra of pyridine hemochromes were used to determine the amount of heme *b* by following the value of the difference between absorbance at 557 nm (reduced) and 540 nm (oxidized) using a difference extinction coefficient of 23.98 nM^-1^.cm^-1^ (6).

1. Caldelari I, Chane-Woon-Ming B, Noirot C, Moreau K, Romby P, Gaspin C, Marzi S. 2017. Complete genome sequence and annotation of the *Staphylococcus aureus* strain HG001. *Genome Announc* 5:e00783-17.

2. Poyart C, Trieu-Cuot P. 1997. A broad-host-range mobilizable shuttle vector for the construction of transcriptional fusions to B-galactosidase in Gram-positive bacteria. *FEMS Microbiology Letters* 156:193-198.

3. Arnaud M, Chastanet A, Debarbouille M. 2004. New vector for efficient allelic replacement in naturally nontransformable, low-GC-content, gram-positive bacteria. *Appl Environ Microbiol* 70:6887-91.

4. Lechardeur D, Cesselin B, Liebl U, Vos MH, Fernandez A, Brun C, Gruss A, Gaudu P. 2012. Discovery of an intracellular heme-binding protein, HrtR, that controls heme-efflux by the conserved HrtB HrtA transporter in *Lactococcus lactis*. *J Biol Chem* 287:4752-4758.

5. Ebner P, Rinker J, Nguyen MT, Popella P, Nega M, Luqman A, Schittek B, Di Marco M, Stevanovic S, Gotz F. 2016. Excreted cytoplasmic proteins contribute to pathogenicity in *Staphylococcus aureus*. *Infect Immun* 84:1672-81.

6. Berry EA, Trumpower BL. 1987. Simultaneous determination of hemes *a*, *b*, and *c* from pyridine hemochrome spectra. *Anal Biochem* 161:1-15.

**Supplemental Table legend**

**Table S1.** (A) Strains and plasmids. (B) List of oligonucleotides.

**Supplemental figure legends**

**Figure S1.** Transient induction of P*_hrtBA_* during stationary and exponential growth phases. (A) Kinetic of P*_hrtBA_* induction in stationary phase. HG001 WT and Δ*hrtBA* strains transformed with pP*_hrtBA_*-GFP. ON culture in CDM were distributed in a 96 well microplate. OD_600_ and GFP expression were followed in a spectrofluorimeter Infinite (Tecan). Results of hemin induced fluorescence minus non-induced (background, 0 µM hemin) are displayed. Results represent the average ± S.D. from triplicate biological samples. (B) Kinetic of HrtB expression following addition of hemin in HG001 WT. Strain from ON culture was diluted to OD_600_ = 0.1 in BHI. 2 µM hemin were added to the culture at t=0. At the indicated time points, samples of bacteria (containing equivalent number of bacteria) were pelleted and OD normalized to 0.5 by resuspending the pellet in PBS. At the indicated time point, samples of bacteria were pelleted and processed for SDS-PAGE and immunoblot with a α-HrtB antibody.

**Figure S2.**  Heme accumulates in HG001 Δ*hrtBA* strain. WT and Δ*hrtBA* strains were grown to OD600 = 1 prior addition of 10 µM hemin in BHI for an additional 1.5 h. (A) Bacteria were pelleted by centrifugation and photographed. (B) Heme content in the cell pellets was determined by the pyridine hemochrome assay on lysates. Background from bacteria non-exposed to hemin was substracted. Results represent the average ± S.D. from biological triplicates. *, P =0.045, Student’s t test.

**Figure S3.** Superimposition of all the docking solutions using the intracellular part of HssS. As compared to Fig 3B using the ECD of HssS, all the docking solutions are above -8 kcal/mol and are scattered on the surface of the protein.

**Figure S4.** WebLogo representation of AAs [1-188] of HssS. 150 AAs sequences that sample the list of homologues to HssS (with a minimum of 35 % identity) were used. Residues targeted by site-directed mutagenesis are represented in orange for phenylalanine and pink for arginine. The AAs corresponding to the predicted ECD are underlined in red, and residues predicted to be within 5Å of the heme are marked with a grey line.

**Figure S5.** Growth of HG001 ∆*hssRS* complemented either with pGFP(HssS) or HssS variants R94A, R163A, F25A, F128A, F165A. All strains were were diluted from an ON preculture to an OD_600_ of 0.01 in CDM and grown in a microplate. OD_600_ was recorded every 20 min for the indicated time in a spectrophotometer (Spark, Tecan). (A) Growth of Δ*hssRS* mutants transformed either with pGFP(HssS), pGFP(HssS R94A), pGFP(HssS R163A), pGFP(HssS R94A, R163A) or empty vector (pØ). Results represent the average ± S.D from triplicate biological samples. (B) Growth of Δ*hssRS* mutants transformed either with pGFP(HssS), pGFP(HssS F25A), pGFP(HssS F128A), pGFP(HssS F165A) or empty vector (pØ). Results of bacterial growth minus medium background are displayed. Results represent the average ± S.D from triplicate biological samples. (C) Growth of Δ*hssRS* mutants transformed either with pGFP(HssS), pGFP(HssS 4 mutA) or empty vector (pØ). Results of bacterial growth minus medium background are displayed. Results represent the average ± S.D from triplicate biological samples.

**Figure S6.** Comparative effect of replacing Arg94, Arg163, Phe25 and Phe128 with Ala or Glu on HssS activation. (A) P*_hrtBA_* transcriptional induction by HssS, HssS R94A, R163A (HssS 2R🡺A), HssS R94E, R163E (HssS 2R🡺E), HssS R94A, R163A, Phe25A, Phe128A (4mutA) and HssS R94E, R163E, Phe25E, Phe128E (4mutE). Kinetics of P*_hrtBA_* induction in HG001 Δ*hssRS* mutant transformed either with pGFP(HssS), pGFP(HssS 2R🡺A), pGFP(HssS 2R🡺E), pGFP(HssS 4mutA) and pGFP(HssS 4mutE). Strains from ON cultures were diluted to OD_600_ = 0.01 in CDM ± 1 µM of hemin in a 96 well microplate. OD_600_ and GFP expression were followed in a spectrofluorimeter Infinite (Tecan) as in Fig. 2. Results of hemin induced fluorescence minus non-induced (background, 0 µM hemin) are displayed. Results represent the average ± S.D. from triplicate biological samples. ***, P <0.001, Student’s t test (for the 4 mutant strains compared to the WT). The corresponding growth curves are shown. (B) Hemin toxicity in HssS, HssS 2R🡺A, HssS 2R🡺E, HssS 4mutA and HssS 4mutE expressing strains. Strains as in (A) were diluted from an ON preculture to an OD_600_ of 0.01 in BHI supplemented with 20 µM hemin and grown in a 96 microplate. OD_600_ was recorded every 20 min for the indicated time in a spectrophotometer (Spark, Tecan). Results represent the average ± S.D from triplicate biological samples. (C, D) Comparative expressions of HssS-HA, HssS-HA 2R🡺A, HssS-HA 2R🡺E, HssS-HA 4mutA, HssS-HA 4mutE and HrtB. Strains as in (A) were diluted in BHI from ON culture at OD_600_ = 0.01. Cultures were supplemented ± 1 µM hemin and grown for 1.5 h. HG001 Δ*hssRS* transformed with the empty plasmid (Ø) was used as a control. HssS-HA and HrtB expression were monitored on bacterial lysates by Western Blot (WB) with an anti-hemagglutinin antibody (α-HA) and an anti-HrtB antibody respectively (α-HrtB). Results are representative of three independent experiments.

**Figure S7.** Purification of HssS- His_6_ WT, F25A, F128A, R94A, R163A, and 4mutA in *E. coli*. Coomassie staining of purified WT, F25A, F128A, R94A, R163A, and 4mutA HssS-His_6_. 5 µg of each protein were processed for SDS-PAGE following nickel affinity chromatography purification and dialysis. The acrylamide gel was stained with Imperial protein stain (ThermoFischer).

**Figure S8.** HssS ΔECD is expressed at the membrane and signals heme detoxification. (A) Cell fractionation of HssS FL and ΔECD expressing HG001 bacteria. Δ*hssRS* HG001 transformed with either pGFP(HssS) or pGFP(HssS ΔECD) were grown in BHI to an OD_600_ = 1, lysed and processed for the separation of membrane from cytoplasm by ultracentrifugation. Whole cell lysate, isolated membrane and cytoplasm enriched fractions were processed for SDS-PAGE and immunoblot using an anti-HA antibody. An anti-GAPDH antibody was used as a marker of cytoplasm. Result is representative of 3 independent experiments. (B-C) Heme toxicity in liquid culture of Δ*hssRS* HG001 transformed with either pGFP(HssS), pGFP(HssS ΔECD) or empty vector (pØ). Bacteria were diluted from ON cultures to an OD_600_ = 0.01 and grown in BHI without (B) or with 10 µM hemin in a microplate spectrophotometer (Spark, Tecan). OD_600_ was recorded every 15 min for the indicated time. Results of bacterial growth minus medium background are displayed. Results represent the average ± S.D from triplicate biological samples.
