# Supplementary figures and images for: HssS activation by membrane heme defines a paradigm for two-component system signaling in Staphylococcus aureus
Source: mBio. 2024 Apr 29;15(6):e00230-24. doi: 10.1128/mbio.00230-24 (PMC11237747; doi:10.1128/mbio.00230-24)

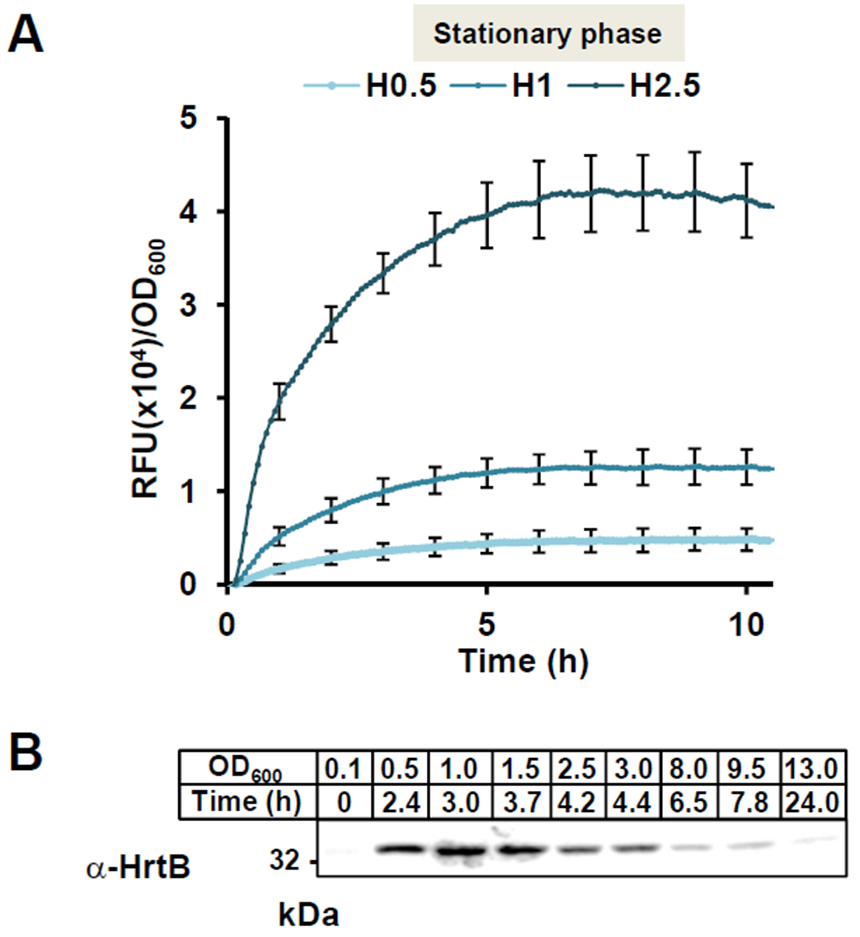

Supplement: Fig. S1 — Transient induction of PhrtBA during stationary and exponential growth phases. [file mbio.00230-24-s0002.tif]

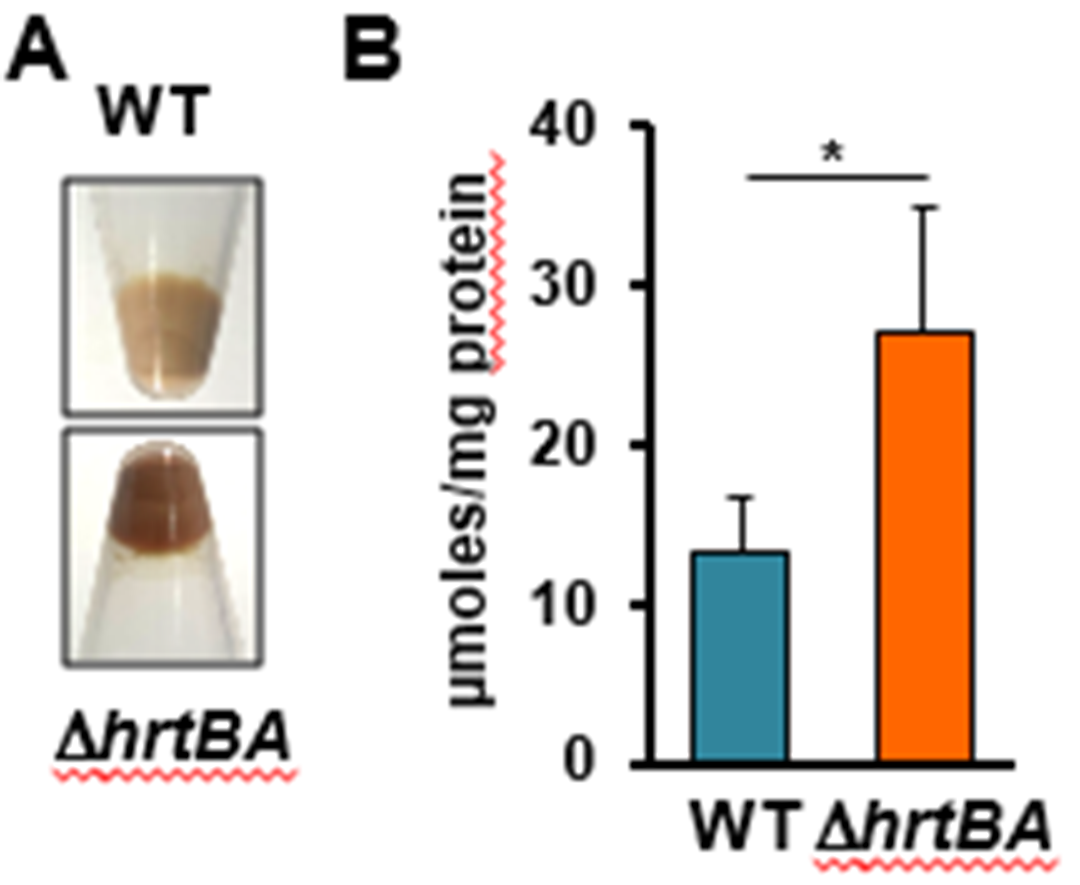

Supplement: Fig. S2 — Heme accumulates in HG001 ΔhrtBA strain. [file mbio.00230-24-s0003.tif]

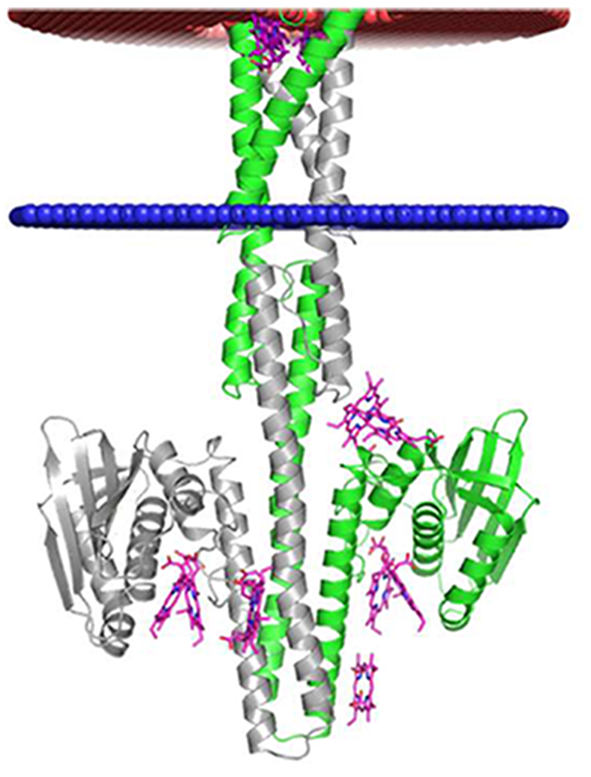

Supplement: Fig. S3 — Superimposition of all the docking solutions using the intracellular part of HssS. [file mbio.00230-24-s0004.tif]

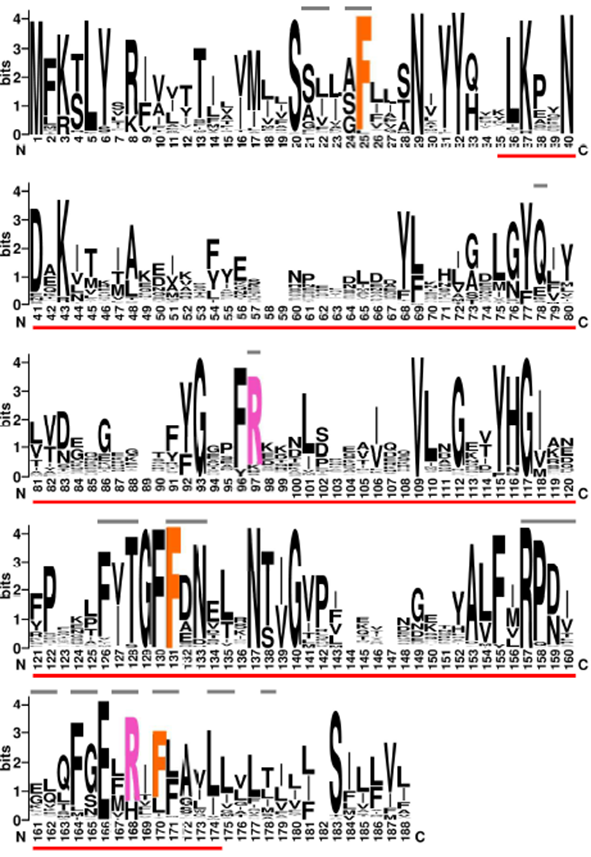

Supplement: Fig. S4 — WebLogo representation of AAs 1-188 of HssS. [file mbio.00230-24-s0005.tif]

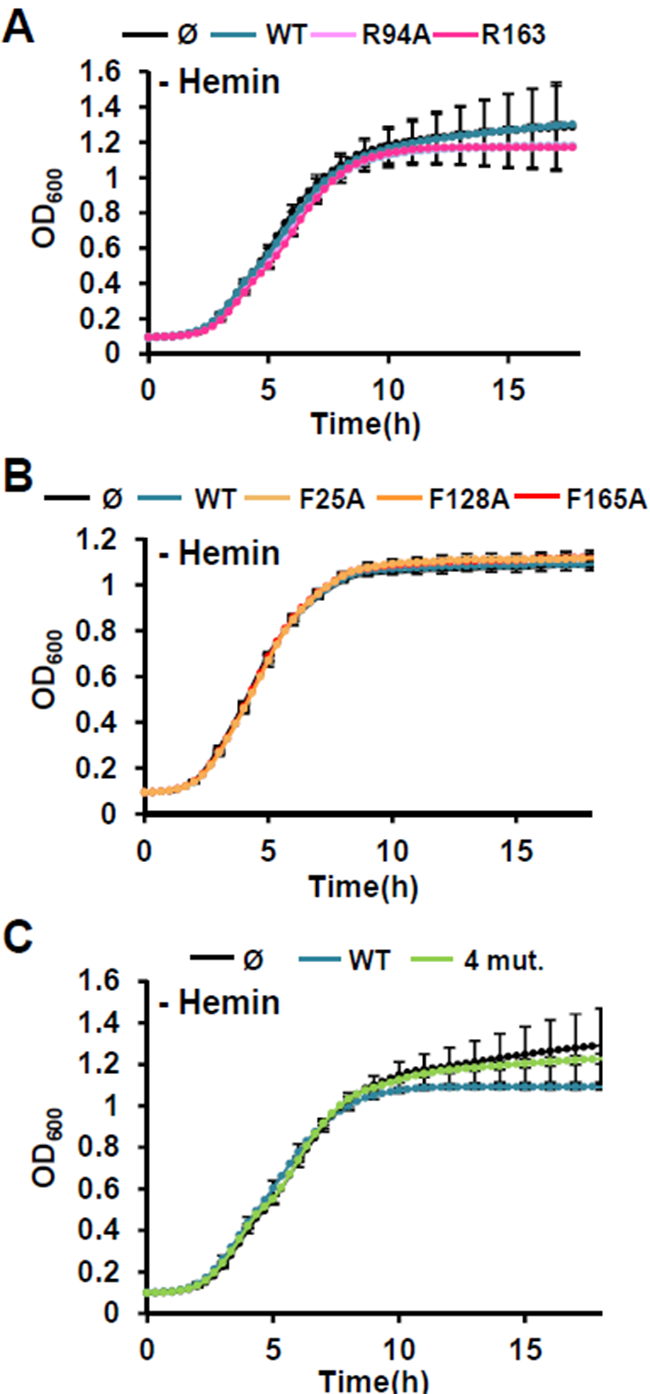

Supplement: Fig. S5 — Growth of HG001 ∆hssRS complemented with pGFP(HssS) or HssS variants. [file mbio.00230-24-s0006.tif]

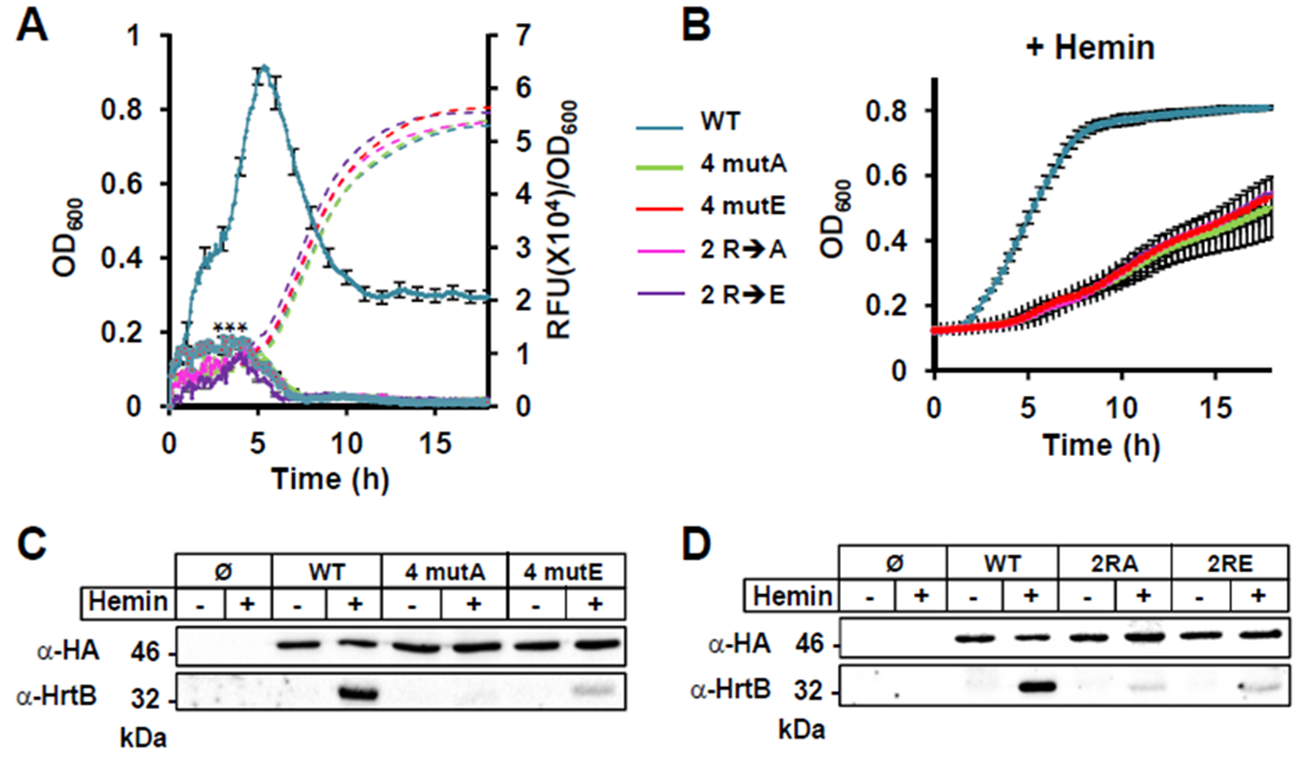

Supplement: Fig. S6 — Comparative effect of replacing Arg94, Arg163, Phe25, and Phe128 with Ala or Glu on HssS activation. [file mbio.00230-24-s0007.tif]

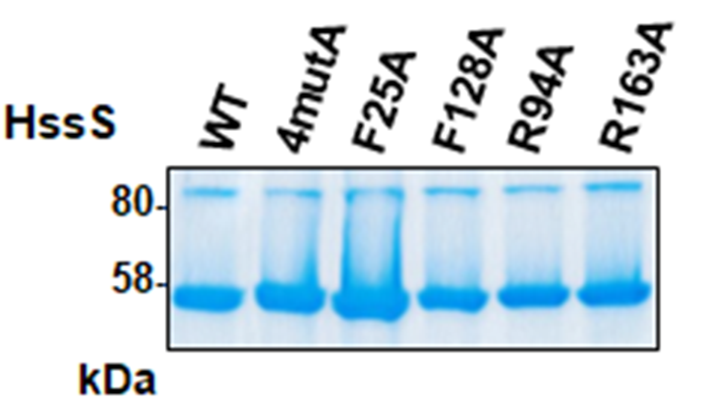

Supplement: Fig. S7 — Purification of HssS and HssS variants from E. coli. [file mbio.00230-24-s0008.tif]

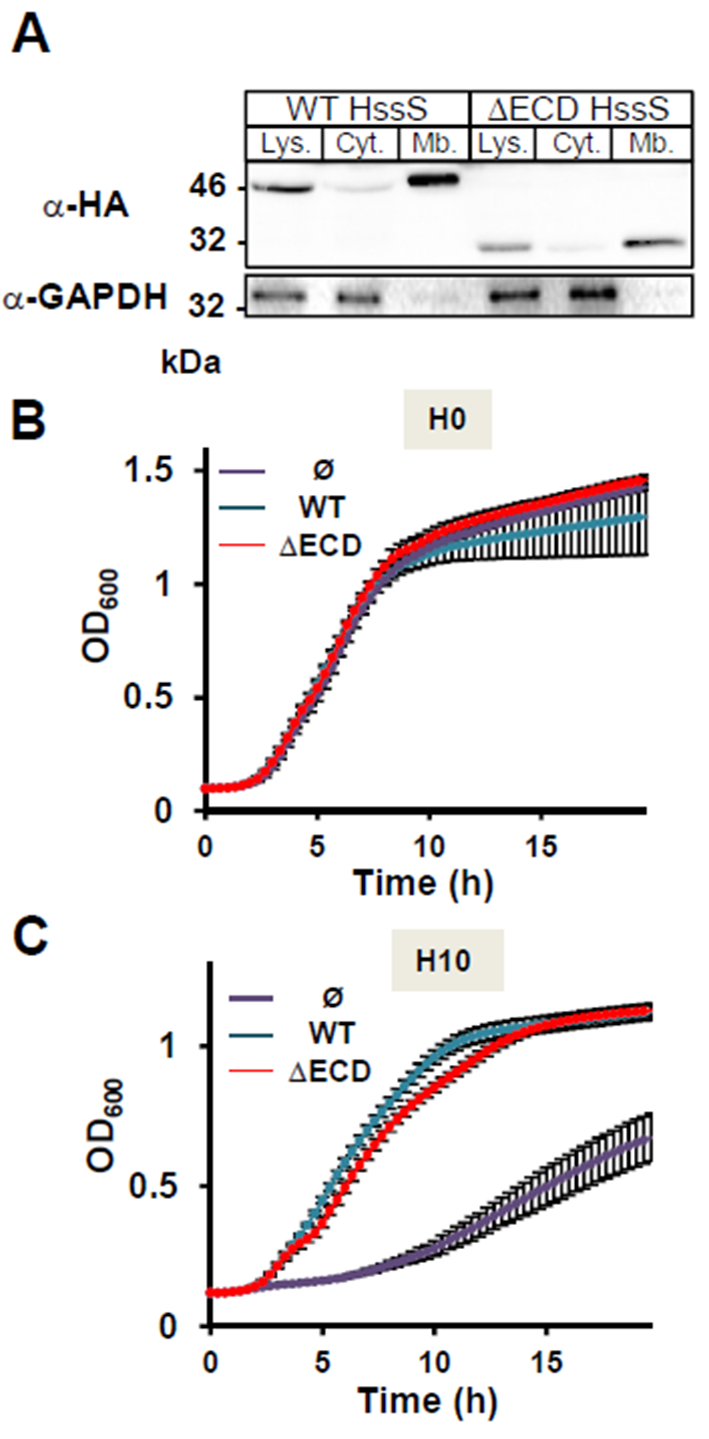

Supplement: Fig. S8 — HssS ΔECD is expressed at the membrane and signals heme detoxification. [file mbio.00230-24-s0009.tif]
