# Supplementary material for: HssS activation by membrane heme defines a paradigm for two-component system signaling in Staphylococcus aureus
Source: mBio. 2024 Apr 29;15(6):e00230-24. doi: 10.1128/mbio.00230-24 (PMC11237747; doi:10.1128/mbio.00230-24)
Supplement: Table S1 — Strains, plasmids, and oligonucleotides. [file mbio.00230-24-s0010.docx]

**Table S1**

(A) Strains and plasmids.

| ***Strain/plasmid*** | ***Characteristics*** | ***Source/ reference*** |
| --- | --- | --- |
| ***Strain***  ***E. coli*** | | |
| TOP10 | *F^-^ mcrA Δ(mrr-hsdRMS-mcrBC) Δ80lacZΔM15 lacX74 recA1 deoR araD139 Δ(ara-leu)7697 galU galK rpsL (Str^r^) endA1* | Invitrogen |
| BL21 (DE3) | *lacI^q^ rrnB T14 ΔlacZWJ16 hsdR514 ΔaraBA-D_AH33_*  *ΔrhaBAD_LD78_* | Invitrogen |
| BL21 C43 (DE3) | A derivative of BL21 (DE3) for the expression of toxic proteins | Merck |
| ***S. aureus*** |  |  |
| RN4220 | *S. aureus* cloning recipient ATCC 8325-4 derivative restriction negative | (1) |
| HG001 | *Staphylococcus aureus* HG001 strain, derivative of the RN1 (NCT8325) strain with restored rbsU (a positive activator of SigB). | (2) |
| HG001H1 | HG001 Δ*hssRS*, deletion of *hssR* and *hssS* genes | (3) |
| HG001H2 | HG001 Δ*hrtBA*, deletion of *hrtB* and *hrtA* genes | This study |
| ***Plasmid*** | | |
| pTCV-*lac* | Conjugative *E. coli* Gram-positive bacteria shuttle plasmid carrying the promoter less *E. coli lacZ* gene for constructing transcriptional fusions. Kan^R^, Ery^R^ | (4) |
| pCN52 | E. coli-S.aureus bacteria shuttle plasmid with gfpmut2 *reporter* construct. Amp^R^, Ery^R^ | (5) |
| pAW8 | Cloning shuttle vector, pMB1 ori for replication in E. coli, pAMα1 ori for replication in gram-positive organisms. Tet^R^, Amp^R^ | (6) |
| pMAD | Cloning shuttle replication-thermosensitive vector for generating stable chromosomal mutations in low G-C Gram positive bacteria. Amp^R^, Ery^R^ | (7) |
| pET2160 | Vector derived from pET21d for the expression of proteins with a six-histidine tag (His_6_) at the C-terminal extremity. | (8) |
| pP*_hrtBA_* -*lac* | *S. aureus* HG001 *hrtBA* promoter region cloned into pTCV-*lac.*  Kan^R^, Ery^R^ | This study |
| pP*_hssRS_*-*lac* | *S. aureus* HG001 *hssRS* promoter region region cloned into pTCV-*lac.*  Kan^R^, Ery^R^ | This study |
| pUC *hssRS*-HA, P*_hrtBA_* | P_hssRS_ promoter region and *hssRS* genes followed by the promoter region of *hrtBA* from *S. aureus* HG001 cloned into pUC*.* Amp^R^ | Proteogenix  (France) |
| p*hssRS-*HA | *hssRS* promoter region and *hssRS* genes with a HA epitope at the C-terminal of *hssS* from *S. aureus* HG001 in pAW8. Tet^R^, Amp^R^ | This study |
| pP*_hrtBA_* -*gfp* | *S. aureus* HG001 *hrtBA* promoter region cloned into pCN52*.*  Amp^R^, Ery^R^ | This study |
| pGFP(HssS) | p*hssRS*-HA, P*_hrtBA_*-*gfp*. Promoter region and *hssRS* genes with a HA epitope at the C-terminal of *hssS* from *S. aureus* HG001 and promoter region of *hrtBA* cloned upstream GFP in pCN52. Amp^R^, Ery^R^ | This study |
| pGFP(HssS T253A) | p*hssRS*-HA T253A, P*_hrtBA_*-*gfp*. pGFP(HssS) plasmid with a mutation of the codon encoding T253 to alanine codon in *hssS.* Amp^R^, Ery^R^ | E-Zyvec  (France) |
| pGFP(HssS R94A) | p*hssRS*-HA R94A, P*_hrtBA_*-*gfp*. pGFP(HssS) plasmid with a mutation of the codon encoding R94 to alanine codon in *hssS.* Amp^R^, Ery^R^ | E-Zyvec  (France) |
| pGFP(HssS R163A) | p*hssRS*-HA R163A, P*_hrtBA_*-*gfp*. pGFP(HssS) plasmid with a mutation of the codon encoding R163 to alanine codon in *hssS.* Amp^R^, Ery^R^ | E-Zyvec  (France) |
| pGFP(HssS 2R🡺A) | p*hssRS*-HA R94A, R163A, P*_hrtBA_*-*gfp*. pGFP(HssS) plasmid with mutations of the codon encoding R94 and R163 to alanine codon in *hssS.* Amp^R^, Ery^R^ | E-Zyvec  (France) |
| pGFP(HssS 2R🡺E) | p*hssRS*-HA R94E, R163E, P*_hrtBA_*-*gfp*. pGFP(HssS) plasmid with mutations of the codon encoding R94 and R163 to alanine codon in *hssS.* Amp^R^, Ery^R^ | E-Zyvec  (France) |
| pGFP(HssS F25A) | p*hssRS*-HA F25A, P*_hrtBA_*-*gfp*. pGFP(HssS) plasmid with a mutation of the codon encoding F25 to alanine codon in *hssS.* Amp^R^, Ery^R^ | E-Zyvec  (France) |
| pGFP(HssS F128A) | p*hssRS*-HA F128A, P*_hrtBA_*-*gfp*. pGFP(HssS) plasmid with a mutation of the codon encoding F128 to alanine codon in *hssS.* Amp^R^, Ery^R^ | E-Zyvec  (France) |
| pGFP(HssS F165A) | p*hssRS*-HA F165A, P_hrtBA_-*gfp*. pGFP(HssS) plasmid with a mutation of the codon encoding F165 to alanine codon in *hssS.* Amp^R^, Ery^R^ | E-Zyvec  (France) |
| pGFP(HssS 4mutA) | p*hssRS*-HA R94A, R163A, F128A, F165A, P*_hrtBA_*-*gfp*. pGFP(HssS) plasmid with mutations of the codon encoding R94, R163, F25, F128 to alanine codon in *hssS.* Amp^R^, Ery^R^ | E-Zyvec  (France) |
| pGFP(HssS 4mutE) | p*hssRS*-HA R94E, R163E, F128E, F165E, P*_hrtBA_*-*gfp*. pGFP(HssS) plasmid with mutations of the codon encoding R94, R163, F25, F128 to alanine codon in *hssS.* Amp^R^, Ery^R^ | E-Zyvec  (France) |
| pGFP(HssS ΔECL) | p*hssRS* ΔECL-HA, P*_hrtBA_*-*gfp*. pGFP(HssS) plasmid with a deletion of the nucleotides corresponding to the AA [50-129] of HssS. Amp^R^, Ery^R^ | This study |
| pΔ*hrtBA* | *hrtBA* fragment cloned into pMAD to obtain the HG001 Δ*hrtBA* mutant. Amp^R^, Ery^R^ | This study |
| p*hssS-his_6_* | *hssS* cloned into pET2160*.* | This study |
| p*hssS* 4mutA-*his_6_* | *hssS* 4mutA cloned into pET2160*.* | This study |
| p*hssS* F25A*-his_6_* | *hssS* F25A cloned into pET2160*.* | This study |
| p*hssS* F128A*-his_6_* | *hssS* F128A cloned into pET2160*.* | This study |
| p*hssS* R94A*-his_6_* | *hssS* R94A cloned into pET2160*.* | This study |
| p*hssS* R163A*-his_6_* | *hssS* R163A cloned into pET2160*.* | This study |

1. Kreiswirth BN, Lofdahl S, Betley MJ, O'Reilly M, Schlievert PM, Bergdoll MS, Novick RP. 1983. The toxic shock syndrome exotoxin structural gene is not detectably transmitted by a prophage. *Nature* 305:709-12.

2. Caldelari I, Chane-Woon-Ming B, Noirot C, Moreau K, Romby P, Gaspin C, Marzi S. 2017. Complete genome sequence and annotation of the *Staphylococcus aureus* Strain HG001. *Genome Announc* 5: e00783-17.

3. Toledo-Arana A, Merino N, Vergara-Irigaray M, Debarbouille M, Penades JR, Lasa I. 2005. *Staphylococcus aureus* develops an alternative, ica-independent biofilm in the absence of the arlRS two-component system. *J Bacteriol* 187:5318-29.

4. Poyart C, Trieu-Cuot P. 1997. A broad-host-range mobilizable shuttle vector for the construction of transcriptional fusions to B-galactosidase in Gram-positive bacteria. *FEMS Microbiology Letters* 156:193-198.

5. Charpentier E, Anton AI, Barry P, Alfonso B, Fang Y, Novick RP. 2004. Novel cassette-based shuttle vector system for gram-positive bacteria. *Appl Environ Microbiol 7*0:6076-85.

6. Wada A, Watanabe H. 1998. Penicillin-binding protein 1 of *Staphylococcus aureus* is essential for growth. *J Bacteriol* 180:2759-65.

7. Arnaud M, Chastanet A, Debarbouille M. 2004. New vector for efficient allelic replacement in naturally nontransformable, low-GC-content, gram-positive bacteria. *Appl Environ Microbiol* 70:6887-91.

8. Barreteau H, Bouhss A, Fourgeaud M, Mainardi JL, Touze T, Gerard F, Blanot D, Arthur M, Mengin-Lecreulx D. 2009. Human- and plant-pathogenic *Pseudomonas* species produce bacteriocins exhibiting colicin M-like hydrolase activity towards peptidoglycan precursors. *J Bacteriol* 191:3657-64.

(B) List of oligonucleotides

| ***Primer*** | ***Sequence 5’-3’*** | ***Target*** |
| --- | --- | --- |
| **O1** | ATTTTAgaattcgcaccatagctataaact | pP*_hrtBA_*-*lac* |
| **O1** | ATTTTAggatccatcgattcacttctccct | pP*_hrtBA_*-*lac* |
| **O3** | ATTTTAGAATTCGATCCATCGATTCACTTC | pP*_hssRS_*-*lac* |
| **O4** | TAAAATGGATCCAGCTATAAACTCCCTTAT | pP*_hssRS_*-*lac* |
| **O5** | TTACCGGAATTCATCGATTCACTTCTCCCT | pP*_hrtBA_*-*gfp* |
| **O6** | AATCGCGGATCCAGCTATAAACTCCCTTAT | pP*_hrtBA_*-*gfp* |
| **O7** | ATAATAGGATCCttaagcataatctggaac | pGFP(HssS) |
| **O8** | TAATAAGGTACCATCGATTCACTTCTCCCT | pGFP(HssS) |
| **O9** | AATATCTGGACGCATATTAGATGCTTTTAA | pGFP(HssS ΔECL) |
| **O10** | TTAAAAGCATCTAATATGCGTCCAGATATT | pGFP(HssS ΔECL) |
| **O11** | CACCTCGTTATTTGAACACTTTGATA | pET200-hrtB ECL |
| **O12** | TTAACTAACAATCATCATATTT | pET200-hrtB ECL |
| **O13** | TTAATTGGATCCACTGTTTCAATTG | pMAD Δ*hrtBA* |
| **O14** | CGTCTTTACAAGCCAATCGATTCACTTCTC | pMAD Δ*hrtBA* |
| **O15** | GAGAAGTGAATCGATTGGCTTGTAAAGACG | pMAD Δ*hrtBA* |
| **O16** | AATTAACCCGGGGTGCCGTCTCAGC | pMAD Δ*hrtBA* |
| **O17** | GGGGCTGCAGCTCCCTATTTCTTCTTTAGCG | p*hssRS*-HA |
| **O18** | AATTAAGGATCCTTAAGCATAATCTGGAACATCATATGGATACAT | p*hssRS*-HA |
| **O19** | TATTATGGATCCATGTTTAAAACACTCATAGCT | p*hssS-his_6_* |
| **O20** | TATTATCTCGAGCATAAGTGAATTATTTGGCAG | p*hssS-his_6_* |
|  |  |  |
